# Nicotinic receptor in dopamine neurons mediates learned odor choice

**DOI:** 10.1371/journal.pgen.1012356

**Published:** 2026-10-29

**Authors:** Nobuhiro Yamagata, Nobuhiro Takahashi, Rino Ichikawa, Eru Wada, Momo Murakami, Ayako Abe, Mai Kanno, Toshiharu Ichinose, Norihiro Katayama, Hiromu Tanimoto

**Affiliations:** 1 Graduate School of Engineering Science, Akita University, Akita, Japan; 2 Graduate School of Life Sciences, Tohoku University, Sendai, Japan; 3 Frontier Research Institute for Interdisciplinary Sciences, Tohoku University, Sendai, Japan; 4 Information Education Center, Shokei Gakuin University, Natori, Japan; Cornell University College of Agriculture and Life Sciences, UNITED STATES OF AMERICA

## Abstract

Nicotinic acetylcholine signaling modulates dopaminergic output in mammalian midbrain circuits and can thereby influence decision-making. In *Drosophila*, dopamine neurons (DANs) in the protocerebral anterior medial (PAM) cluster convey sugar reward signals and promote approach toward a rewarded odor after associative learning. Although nicotinic acetylcholine receptors (nAChRs) have been implicated in regulating PAM DAN excitability and transmitter release, how this modulation supports specific cognitive operations during learning and memory remains unclear. Here, we tested the role of nAChRs in PAM DANs in odor–sugar associative learning and memory. Among nine nAChR genes examined, loss of the *nicotinic acetylcholine receptor α1 subunit* (*Dα1*) in PAM neurons impaired appetitive memory. Consistent with this requirement, *Dα1* was detected in these DANs at both the transcript and protein levels, with *Dα1* puncta observed in PAM axon-terminal and putative dendritic regions. Importantly, live imaging showed that *Dα1* knockdown in PAM DANs attenuated odor-evoked synaptic Ca^2+^ signals, whereas sugar-evoked Ca^2+^ responses were largely preserved. Behaviorally, *Dα1* knockdown in PAM DANs reduced performance in a two-odor forced-choice discrimination task, whereas no performance deficit was detected across the single-odor pursuit conditions or innate odor choice. Together, these results identify a nicotinic mechanism in PAM DANs that supports odor-evoked synaptic activity and contributes to learned odor-choice performance when competing odor cues are present.

## Author summary

Acetylcholine (ACh) and dopamine (DA) are crucial neurotransmitters that form an important axis for cognitive functions. Understanding their roles in decision-making is of utmost interest, as alterations in this axis are linked to the pathophysiology of psychiatric disorders, including nicotine addiction and schizophrenia. This study focuses on the nicotinic acetylcholine receptor, specifically the *Drosophila* α1 (*Dα1*) subunit found in rewarding dopamine neurons of fruit flies. Disrupting *Dα1* reduced odor-evoked synaptic activity in reward-related dopamine neurons and impaired learned odor choice when flies were required to choose between competing odors. These findings indicate a vital role for *Dα1* in reward circuits that support value-based decision-making and provide a tractable model of the ACh-DA axis for linking cellular actions to behavior.

## Introduction

Dopamine neurons (DANs) integrate synaptic and neuromodulatory signals. Their outputs vary with afferent connectivity and with receptor type and localization, which together support integration of multisensory cues and internal state [1–3]. Across phyla, nicotinic acetylcholine receptors (nAChRs) regulate dopaminergic output through presynaptic and somatodendritic mechanisms [4–7]. This regulation forms the ACh–DA axis, linking cholinergic drive to dopaminergic signaling across cellular compartments and circuit nodes. Distinct cholinergic sources engage nAChRs on DAN axons [8–11]. In turn, they tune DAN excitability and moment-to-moment DA release, supporting DAN functions in reward, learning, motivation, and action [8,12]. Disruption of nicotinic control is also implicated in movement and neuropsychiatric disorders [13–15], underscoring its critical role in proper DAN functionality [16,17].

*Drosophila* learn to associate odors with sugar. The memory forms in the mushroom body (MB) [18–22], where sparse ensembles of the ~ 2,000 intrinsic Kenyon cells (KCs), which are cholinergic, encode odors [23–27]. A subset of protocerebral anterior medial (PAM) DANs conveys sugar-reward signals to the MB [28,29]. Approximately 15 anatomically defined PAM subtypes tile the MB lobes, and their functions are shaped by the MB’s compartmental organization. Across 15 MB compartments, each PAM subtype interacts with KCs within its target compartment and modulates KC-to-MBON synapses, while MB output neurons (MBONs) provide valence-coding outputs [30–32]. PAM subtypes receive distinct afferents and engage microcircuits within and outside the MB, further diversifying the function [33,34]. These circuit motifs employ different neurotransmitters and receptor complements [35], providing structural substrates for reinforcement signaling [28,29,36–39], valence-based odor pursuit [40–43] both at cellular [44–46] and subcellular resolution [47,48].

Acetylcholine is the main fast transmitter in the fly brain [49,50] and is used by major olfactory pathways, including projection neurons (PNs), MBKCs, subsets of lateral horn output neurons, and some MBONs [35,51–54]. Ultrastructural reconstructions reveal many cholinergic synapses from KCs and MBONs onto PAM terminals and dendrites [31,32,55], whereas direct PN input to PAM dendrites is limited [54]. Consistent with this anatomy, *in vivo* and *ex vivo* measurements have shown that acute ACh stimulation evokes rapid dopamine release in the adult *Drosophila* MB through nicotinic signaling [4,56–58]. Single-cell transcriptomic and genetic data further indicate that PAM neurons express multiple nAChR subunit transcripts [44], supporting a role for cholinergic control of PAM DANs during memory-related processing. Despite these conserved circuit features and cross-species evidence, however, a clear cognitive account of nicotinic regulation in reward-related DANs remains elusive.

This study examined how nAChRs in PAM DANs contribute to appetitive learning in flies. Among the nAChR genes tested, loss of the nicotinic acetylcholine receptor α1 (*Dα1*) in PAM neurons selectively impaired appetitive memory. Dα1 was expressed in these DANs, and its knockdown attenuated odor-evoked Ca^2+^ responses, whereas sugar-evoked responses were largely preserved. Behaviorally, Dα1 knockdown in PAM DANs reduced performance in a forced two-odor discrimination task. Together, these results identify a nicotinic mechanism within reward-related PAM DANs that supports odor-evoked activity and contributes to learned odor-choice performance when flies choose between competing odor cues during memory-guided behavior.

## Results

### Dα1 is required in the PAM DANs for appetitive memory

To clarify the functional significance of cholinergic regulation of dopaminergic signaling, we examined how nicotinic receptors regulate PAM dopaminergic neurons (PAM DANs). In *Drosophila*, the nAChR repertoire comprises ten α/β subunit genes, facilitating a systematic genetic screen [59–66]. Using transgenic RNAi driven by *R58E02-GAL4* [29], we knocked down each nAChR subunit in PAM neurons. Knockdown of two subunits, *Dα1* and *Dα5*, reduced immediate memory in the initial RNAi screen (Fig 1A). We therefore tested both candidates with full genetic controls. The *Dα1* knockdown phenotype was confirmed in this analysis, whereas the apparent *Dα5* effect was not supported when the corresponding genetic controls were included (Fig 1B). Adult-specific pan-neuronal expression of the same RNAi construct significantly reduced *Dα1* transcript levels, as measured by qRT-PCR (S1 Fig), validating the efficacy of the RNAi reagent. To test *Dα1* function using an independent genetic strategy, we disrupted *Dα1* in PAM neurons by expressing *Cas9* with *R58E02-GAL4* together with *Dα1 sgRNA*. PAM-directed *Dα1* disruption impaired appetitive memory, similar to *Dα1* RNAi (Fig 1C). We therefore focused on *Dα1* in the subsequent experiments. *Dα1* knockdown did not affect basic sensory perception (odor avoidance and sugar attraction; Fig 1D-1F), suggesting that cholinergic input to PAM DANs is required predominantly for memory processes.

**Fig 1:**
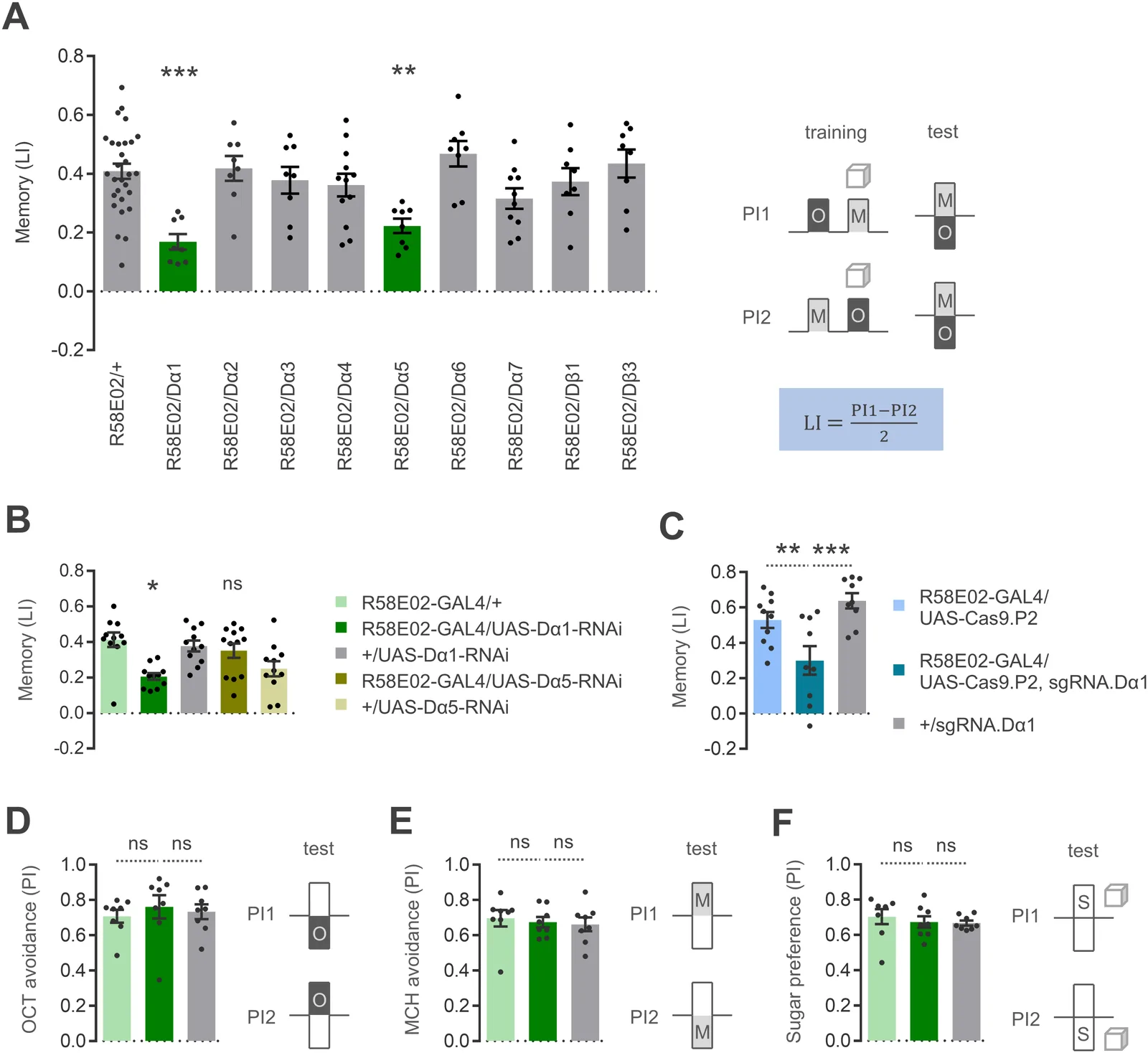
Cholinergic regulation of appetitive dopamine neurons for sugar learning. **(A)** RNAi screen for nicotinic receptor subtypes required in PAM neurons for appetitive memory. Bars indicate mean ± SEM. **p < 0.01, ***p < 0.001, Dunnett’s multiple comparisons test; N = 29, 8, 8, 8, 12, 8, 8, 10, 8, and 8. **(B)** Full genetic controls for Dα1 and Dα5 knockdown in PAM neurons. Dα1 knockdown impaired appetitive memory, whereas the Dα5 effect was not confirmed with genetic controls. *p < 0.05; ns, not significant; Dunn’s multiple comparisons test; N = 11, 11, 11, 12, and 11. **(C)** PAM-specific conditional knockout of Dα1 using R58E02-GAL4-driven Cas9 and Dα1 sgRNA impaired appetitive memory. **p < 0.01, ***p < 0.001; Holm-Sidak’s multiple comparisons test; N = 10, 9, and 9. **(D-F)** Sensory-control assays. Dα1 knockdown did not significantly affect 3-OCT avoidance **(D)**, 4MCH avoidance **(E)**, or sugar preference **(F)**. ns, not significant; N = 8, 8, and 8 for each assay.

### Expression of *Dα1* in PAM DANs

To determine *Dα1* expression in the PAM neurons that supports appetitive memory, we first examined *Dα1* transcriptional reporter expression using *nAChRα1-2A-GAL4 [*67*]* together with *MB247-GAL80* [68] to reduce KCs labeling. This labeling revealed GFP-positive somata in the anatomical PAM soma region and neurites in MB lobe regions associated with PAM dopamine neuron projections (Fig 2A–2C). Because MB compartments contain neurites from multiple neuronal classes, however, this reporter pattern alone cannot assign Dα1 expression specifically to PAM dopamine neurons.

**Fig 2:**
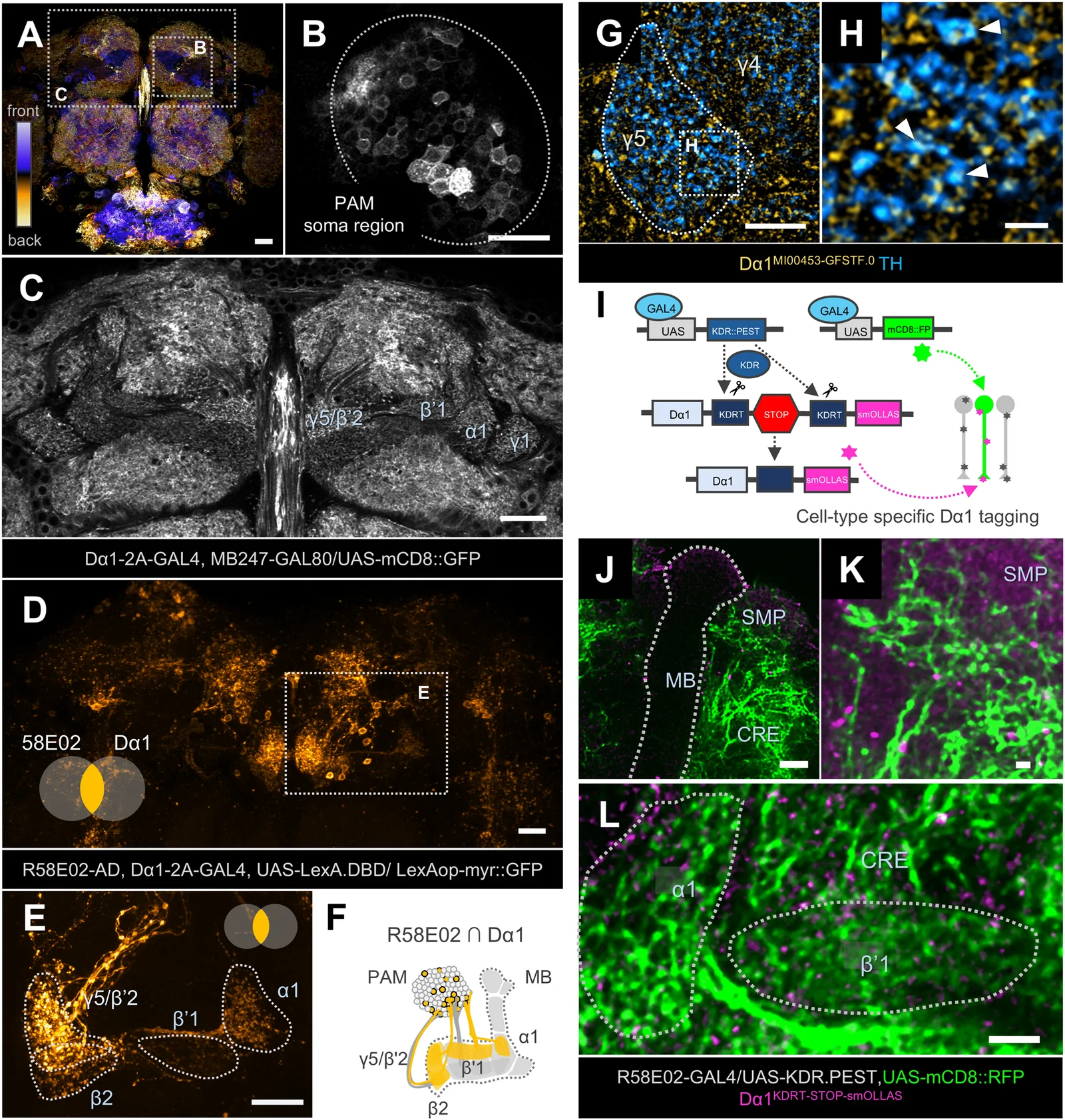
Dα1 expression in PAM DANs. **(A)** Broad protocerebral view of Dα1-2A-GAL4, MB247-GAL80/UAS-mCD8::GFP labeling. The boxed regions indicate the PAM soma region shown in B and the MB lobe region shown in **C. (B)** Magnified optical section of the boxed PAM soma region in **A.** Dashed outlines indicate the anatomical PAM soma regions. **(C)** Magnified view of the boxed MB lobe region in A, showing labeled neurites in MB compartments associated with PAM dopamine neuron projections. **(D, E)** Intersectional labeling of neurons with both R58E02/PAM enhancer activity and Dα1 transcriptional reporter activity. GFP expression depends on the intersection between R58E02-p65.AD and Dα1-2A-GAL4-driven UAS-LexA.DBD. The boxed region in D is magnified in **E. (F)** Schematic summary of the R58E02 ∩ Dα1 intersectional labeling pattern in PAM-associated MB compartments. **(G)** Endogenous Dα1 MiMiC labeling and anti-TH staining in the MB γ-lobe region. The γ5 region is indicated by a white dashed outline, and the boxed region is magnified in **H. (H)** Magnified view of the boxed γ5 region in **G.** White arrowheads indicate regions of spatial overlap between Dα1 and TH signals. **(I)** Schematic of the cell-type-restricted Dα1-smOLLAS labeling strategy. PAM-directed KDR.PEST removes the KDRT-flanked STOP cassette from the Dα1KDRT-STOP-smOLLAS allele, enabling anti-smOLLAS detection of endogenous Dα1-smOLLAS in KDR-expressing PAM neurons. **(J–L)** Cell-type-restricted visualization of endogenous Dα1-smOLLAS in PAM neurons. Dα1-smOLLAS puncta were detected with anti-smOLLAS staining. J and K show MB-extrinsic PAM neurites in the crepine (CRE) and superior medial protocerebrum (SMP), corresponding to putative dendritic/input-enriched regions. L shows PAM axon-terminal regions within MB lobes. Scale bars, 20 µm **(A-E)**, 10 µm **(G)**, 2 µm **(H)**, 10 µm **(J)**, 2 µm (K) and 5 µm **(L)**, respectively.

We therefore used an intersectional strategy to identify neurons with both *R58E02*/PAM enhancer activity and *Dα1* transcriptional reporter activity. In this experiment, GFP expression depended on the intersection between *R58E02-p65.AD* and *Dα1-2A-GAL4*-driven *UAS-LexA.DBD*. This intersectional labeling revealed GFP-positive somata in the PAM region and neurites in MB compartments innervated by reward-related PAM neurons (Fig 2D, 2E). A schematic summary of this anatomical interpretation is shown in Fig 2F. These results support *Dα1* transcriptional reporter activity in specific PAM cell classes.

We next examined Dα1 protein expression using MiMiC protein trap technology [69]. To relate Dα1 protein signal to PAM neurite, we focused on the gamma tip of the MB lobe, which is innervated by specific PAM DAN subclasses. PAM neuron terminals were labeled with an antibody against tyrosine hydroxylase (TH). The two signals spatially overlapped within the MB lobe (Fig 2G, 2H), consistent with Dα1 localization in a PAM terminal region. Because this regional overlap alone cannot assign individual Dα1 puncta to PAM neurites, we next examined endogenous Dα1 protein using a cell-type-restricted smOLLAS-tagging strategy [70]. PAM-directed KDR.PEST expression removes the KDRT-flanked STOP cassette from the Dα1-KDRT-STOP-smOLLAS allele, enabling anti-smOLLAS detection of endogenous Dα1 in KDR-expressing PAM neurons. Using this approach, we detected Dα1-smOLLAS puncta in PAM neurite regions inside and outside the MB lobes (Fig 2J–2L). We cross-referenced these anatomical observations with the adult *Drosophila* hemibrain connectome. Synapse-level analysis of PAM classes corresponding to the γ5, β′2, β′1, and α1 regions analyzed here showed predicted and curated cholinergic inputs both within MB lobe regions and in MB-lobe-external regions, including CRE and SMP (S2 Table). These connectome data provide anatomical support for cholinergic input routes to the PAM neuron classes examined in this study. Together, these complementary approaches support *Dα1* expression in appetitive-memory-relevant PAM classes and provide multiple lines of evidence for Dα1 localization in regions occupied by PAM neurites.

### *Dα1* mediates odor responses in the dopaminergic terminals

Because Dα1-associated puncta were detected in PAM terminal regions and putative dendritic regions, we next asked whether *Dα1* contributes to stimulus-evoked activity in PAM terminals. To resolve the role of *Dα1*, we measured synaptic activity in PAM terminals using the ratiometric active-zone Ca^2+^ sensor *GCaMP6s.brpS.mCherry* [48,71]. Odor and sugar stimulation elicited compartment-specific activity patterns across PAM terminals (Fig 3). In control flies, odor stimulation produced clear responses in the β′1 and α1 terminals, whereas γ5 terminals showed weak or no reliably detectable odor-evoked response. By contrast, sugar presentation evoked responses in the α1 and γ5 terminals, whereas β′1 terminals did not show a significant excitatory sugar response and showed at most a small non-significant decrease.

**Fig 3:**
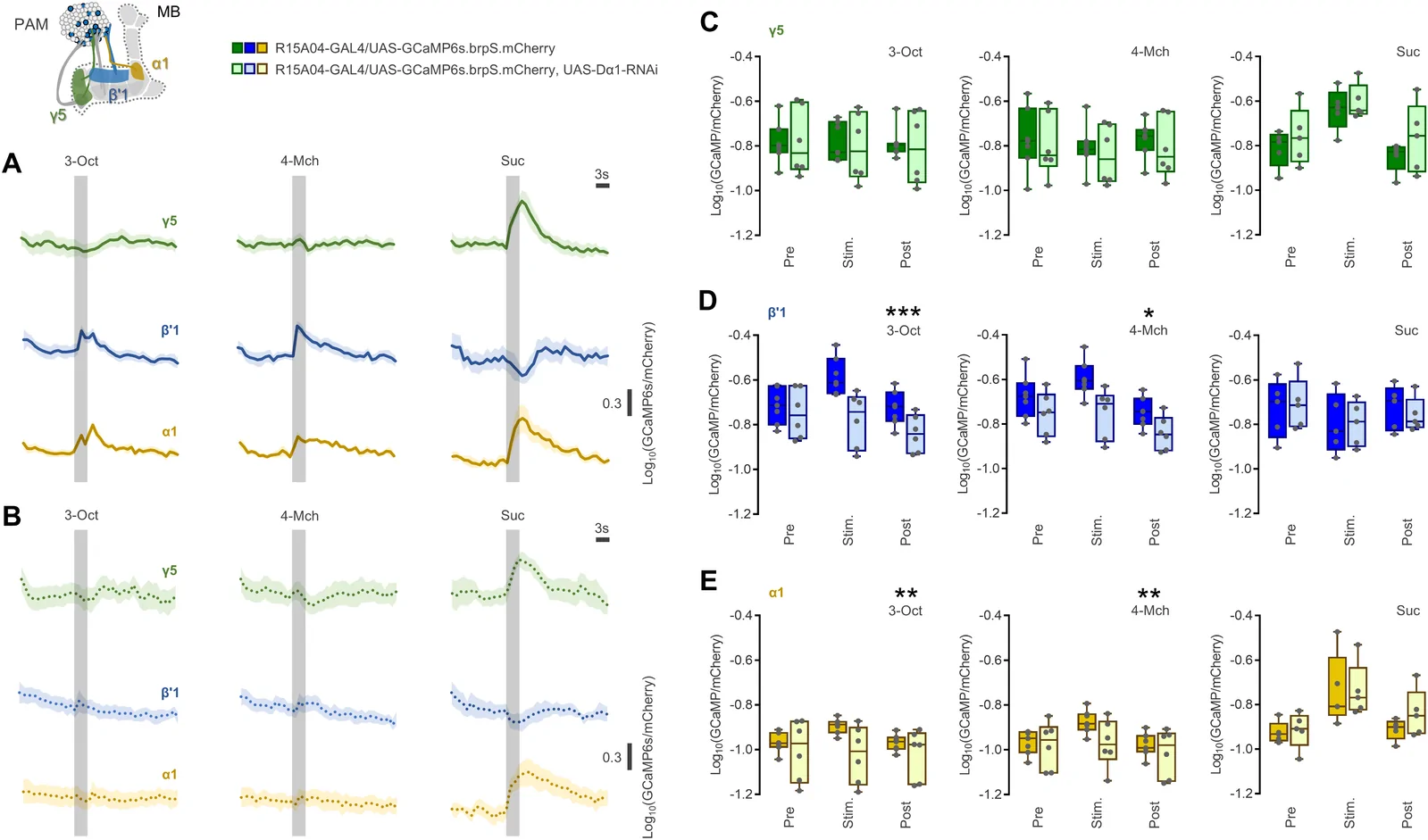
Dα1-mediated odor response in the PAM terminals. **(A, B)** Time course of odor-and sugar-evoked active-zone Ca^2+^ transient of PAM-γ5, PAM-β′1, and PAM-α1 terminals in control (WT; A) and Dα1 knock-down (Dα1-KD; B) flies. Gray shading indicates stimulation for 3 **s.** Traces show mean ± SEM, N = 7 (WT/odor), 6 (Dα1-KD/odor), 5 (WT/sugar), 5 (Dα1-KD/sugar). Scale bars, 3s and 0.3 log_10_(GCaMP6s/mCherry). **(C-E)** Mean log_10_(GCaMP6s/mCherry) signals during pre-stimulus, stimulus, and post-stimulus epochs in PAM-γ5 **(C)**, PAM-β′1 **(D)**, and PAM-α1 (**E**) terminals. Values from the same biological replicates are shown across the pre-stimulus, stimulus, and post-stimulus epochs. Asterisks indicate significant genotype × epoch interactions (*p < 0.05, **p < 0.01, ***p < 0.001; two-way repeated-measures ANOVA). Sidak-adjusted genotype comparisons within individual epochs and complete statistical results are provided in Table 1.

We next compared these responses after *Dα1* knockdown in PAM neurons. *Dα1* knockdown significantly attenuated odor-evoked responses in β′1 and α1 terminals, as indicated by genotype × epoch interactions across pre-stimulus, stimulus, and post-stimulus periods (Fig 3D, 3E). Post hoc genotype comparisons during the stimulus epoch were significant for both odors in β′1 terminals and for 3-OCT, but not 4MCH, in α1 terminals (Table 1). In contrast, we did not detect a genotype-dependent reduction in sugar-evoked responses in the compartments that responded to sugar, including γ5 and α1 terminals (Fig 3C, 3E). These results indicate that *Dα1* is required for odor-evoked synaptic activity in specific PAM terminals, whereas sugar-evoked responses are largely preserved under these conditions.

**Table 1:** List of fly strains and crosses for experiments, and the statistical results.

Figure	Group code	Males	Females	*N*	*D’Agostino normality test* *(P value)*	*Brown-Forsythe test (P value)*	*ANOVA /* *Kruskal-Wallis test* *(P value)*	*Statistical comparison* *(P value or adjusted P value)*
Fig 1A	*1*	*w*	*R58E02-GAL4*	29	*0.9215*	*0.8218*	*One-way ANOVA* < 0.0001 ***	< 0.0001 (1 vs. 2) *** *0.9997 (1 vs. 3)* *0.9935 (1 vs. 4)* *0.8532 (1 vs. 5)* 0.0025 (1 vs. 6) ** *0.7944 (1 vs. 7)* *0.2400 (1 vs. 8)* *0.9878 (1 vs. 9)* *0.9969 (1 vs. 10)* *Dunnett’s test*
	*2*	*UAS-Dα1-RNAi*	*R58E02-GAL4*	8	*0.1926*			
	*3*	*UAS-Dα2-RNAi*	*R58E02-GAL4*	8	*0.3034*			
	*4*	*UAS-Dα3-RNAi*	*R58E02-GAL4*	8	*0.5793*			
	*5*	*UAS-Dα4-RNAi*	*R58E02-GAL4*	12	*0.8861*			
	*6*	*UAS-Dα5-RNAi*	*R58E02-GAL4*	8	*0.3328*			
	*7*	*UAS-Dα6-RNAi*	*R58E02-GAL4*	8	*0.9389*			
	*8*	*UAS-Dα7-RNAi*	*R58E02-GAL4*	10	*0.8681*			
	*9*	*UAS-Dβ1-RNAi*	*R58E02-GAL4*	8	*0.8674*			
	*10*	*UAS-Dβ3-RNAi*	*R58E02-GAL4*	8	*0.4826*			
Fig 1B	*1*	*w*	*R58E02-GAL4*	11	0.0007 ***	*NA*	*Kruskal-Wallis test* 0.0013 **	0.0011 (1 vs. 2) ** 0.0131 (2 vs. 3) * *0.9439 (1 vs. 4)* *0.4697 (4 vs. 5)* *Dunn’s test*
	*2*	*UAS-Dα1-RNAi*	*R58E02-GAL4*	11	*0.8098*			
	*3*	*UAS-Dα1-RNAi*	*w*	11	*0.713*			
	*4*	*UAS-Dα5-RNAi*	*R58E02-GAL4*	12	*0.316*			
	*5*	*UAS-Dα5-RNAi*	*w*	11	*0.8251*			
Fig 1C	*1*	*R58E02-GAL4, UAS-Cas9.P2*	*+*	*10*	*0.6328*	*0.0798*	*One-way ANOVA* 0.0015 **	0.0094 (1 vs. 2) ** 0.0009 (2 vs. 3) *** *Holm-Sidak’s test*
	*2*	*R58E02-GAL4, UAS-Cas9.P2*	*sgRNA.Dα1*	*9*	*0.3894*			
	*3*	*+*	*sgRNA.Dα1*	*9*	*0.5404*			
Fig 1D	*1*	*w*	*R58E02-GAL4*	*8*	0.0191 *	*NA*	*Kruskal-Wallis test* *0.3877*	*0.3816 (1 vs. 2)* *0.6104 (2 vs. 3)* *Dunn’s test*
	*2*	*UAS-Dα1-RNAi*	*R58E02-GAL4*	*8*	0.0058 **			
	*3*	*UAS-Dα1-RNAi*	*w*	*8*	*0.7035*			
Fig 1E	*1*	*w*	*R58E02-GAL4*	*8*	0.0011 **	*NA*	*Kruskal-Wallis test* *0.4350*	*0.5777 (1 vs. 2)* *0.9999 (2 vs. 3)* *Dunn’s test*
	*2*	*UAS-Dα1-RNAi*	*R58E02-GAL4*	*8*	*0.5438*			
	*3*	*UAS-Dα1-RNAi*	*w*	*8*	*0.9155*			
Fig 1F	*1*	*w*	*R58E02-GAL4*	*8*	0.0129 *	*NA*	*Kruskal-Wallis test* *0.3004*	*0.4587 (1 vs. 2)* *0.9999 (2 vs. 3)* *Dunn’s test*
	*2*	*UAS-Dα1-RNAi*	*R58E02-GAL4*	*8*	*0.8461*			
	*3*	*UAS-Dα1-RNAi*	*w*	*8*	*0.3685*			
Fig 3C (3-Oct)	*1 (WT)*	*R15A04-GAL4*	*UAS-GCaMP6s.brpS.mCherry*	*7*	*NA*	*NA*	*Two-way repeated-measures ANOVA* *0.6015 (interaction)* *0.7087 (epoch)* *0.8129 (genotype)*	*0.9999 (pre 1 vs. 2)* *0.9779 (stim 1 vs. 2)* *0.9748 (post 1 vs. 2)* *Sidak’s test*
	*2 (Dα1-KD)*	*R15A04-GAL4*	*UAS-GCaMP6s.brpS.mCherry;UAS-Dα1-RNAi*	*6*	*NA*			
Fig 3C (4-Mch)	*1 (WT)*	*R15A04-GAL4*	*UAS-GCaMP6s.brpS.mCherry*	*7*	*NA*	*NA*	*Two-way repeated-measures ANOVA 0.9154 (interaction)* *0.0829 (epoch)* *0.6163 (genotype)*	*0.9772 (pre 1 vs. 2)* *0.9416 (stim 1 vs. 2)* *0.9139 (post 1 vs. 2)* *Sidak’s test*
	*2 (Dα1-KD)*	*R15A04-GAL4*	*UAS-GCaMP6s.brpS.mCherry;UAS-Dα1-RNAi*	*6*	*NA*			
Fig 3C (Suc)	*1 (WT)*	*R15A04-GAL4*	*UAS-GCaMP6s.brpS.mCherry*	*5*	*NA*	*NA*	*Two-way repeated-measures ANOVA 0.5428 (interaction)* < 0.0001 (epoch) *** *0.3869 (genotype)*	*0.8181 (pre 1 vs. 2)* *0.9471 (stim 1 vs. 2)* *0.5370 (post 1 vs. 2)* *Sidak’s test*
	*2 (Dα1-KD)*	*R15A04-GAL4*	*UAS-GCaMP6s.brpS.mCherry;UAS-Dα1-RNAi*	*5*	*NA*			
Fig 3D (3-Oct)	*1 (WT)*	*R15A04-GAL4*	*UAS-GCaMP6s.brpS.mCherry*	*7*	*NA*	*NA*	Two-way repeated-measures ANOVA 0.0001 (interaction) *** < 0.0001 (epoch) *** 0.0383 (genotype) *	*0.8932 (pre 1 vs. 2)* 0.0018 (stim 1 vs. 2) ** *0.1021 (post 1 vs. 2)* *Sidak’s test*
	*2 (Dα1-KD)*	*R15A04-GAL4*	*UAS-GCaMP6s.brpS.mCherry;UAS-Dα1-RNAi*	*6*	*NA*			
Fig 3D (4-Mch)	*1 (WT)*	*R15A04-GAL4*	*UAS-GCaMP6s.brpS.mCherry*	*7*	*NA*	*NA*	Two-way repeated-measures ANOVA 0.0245 (interaction) * < 0.0001 (epoch) *** 0.0364 (genotype) *	*0.3101 (pre 1 vs. 2)* 0.0108 (stim 1 vs. 2) * *0.1359 (post 1 vs. 2)* *Sidak’s test*
	*2 (Dα1-KD)*	*R15A04-GAL4*	*UAS-GCaMP6s.brpS.mCherry;UAS-Dα1-RNAi*	*6*	*NA*			
Fig 3D (Suc)	*1 (WT)*	*R15A04-GAL4*	*UAS-GCaMP6s.brpS.mCherry*	*5*	*NA*	*NA*	*Two-way repeated-measures ANOVA 0.4744 (interaction)* 0.0078 (epoch) ** *0.9521 (genotype)*	*0.9876 (pre 1 vs. 2)* *0.9999 (stim 1 vs. 2)* *0.9536 (post 1 vs. 2)* *Sidak’s test*
	*2 (Dα1-KD)*	*R15A04-GAL4*	*UAS-GCaMP6s.brpS.mCherry;UAS-Dα1-RNAi*	*5*	*NA*			
Fig 3E (3-Oct)	*1 (WT)*	*R15A04-GAL4*	*UAS-GCaMP6s.brpS.mCherry*	*7*	*NA*	*NA*	Two-way repeated-measures ANOVA 0.0017 (interaction) ** 0.0205 (epoch) * *0.1731 (genotype)*	*0.8896 (pre 1 vs. 2)* 0.0446 (stim 1 vs. 2) * *0.7064 (post 1 vs. 2)* *Sidak’s test*
	*2 (Dα1-KD)*	*R15A04-GAL4*	*UAS-GCaMP6s.brpS.mCherry;UAS-Dα1-RNAi*	*6*	*NA*			
Fig 3E (4-Mch)	*1 (WT)*	*R15A04-GAL4*	*UAS-GCaMP6s.brpS.mCherry*	*7*	*NA*	*NA*	Two-way repeated-measures ANOVA 0.0065 (interaction) ** < 0.0001 (epoch) *** *0.2835 (genotype)*	*0.9873 (pre 1 vs. 2)* *0.1150 (stim 1 vs. 2)* *0.8260 (post 1 vs. 2)* *Sidak’s test*
	*2 (Dα1-KD)*	*R15A04-GAL4*	*UAS-GCaMP6s.brpS.mCherry;UAS-Dα1-RNAi*	*6*	*NA*			
Fig 3E (Suc)	*1 (WT)*	*R15A04-GAL4*	*UAS-GCaMP6s.brpS.mCherry*	*5*	*NA*	*NA*	*Two-way repeated-measures ANOVA 0.4966 (interaction)* < 0.0001 (epoch) *** *0.6173 (genotype)*	*0.9977 (pre 1 vs. 2)* *0.9999 (stim 1 vs. 2)* *0.6191 (post 1 vs. 2)* *Sidak’s test*
	*2 (Dα1-KD)*	*R15A04-GAL4*	*UAS-GCaMP6s.brpS.mCherry;UAS-Dα1-RNAi*	*5*	*NA*			
Fig 4A	*1*	*w*	*R58E02-GAL4*	*16*	*0.2787*	*0.6746*	*One-way ANOVA* 0.0063 **	0.0487 (1 vs. 2) * 0.0041 (2 vs. 3) ** *Sidak’s test*
	*2*	*UAS-Dα1-RNAi*	*R58E02-GAL4*	*16*	*0.6899*			
	*3*	*UAS-Dα1-RNAi*	*w*	*16*	*0.6312*			
Fig 4B	*1*	*w*	*R58E02-GAL4*	*15*	*0.2237*	*NA*	*Kruskal-Wallis test* *0.3654*	*> 0.9999 (1 vs. 2)* *0.3122 (2 vs. 3)* *Dunn’s test*
	*2*	*UAS-Dα1-RNAi*	*R58E02-GAL4*	*16*	0.0063 **			
	*3*	*UAS-Dα1-RNAi*	*w*	*16*	*0.176*			
Fig 4C	*1*	*w*	*R58E02-GAL4*	*8*	*0.7322*	*0.7146*	*One-way ANOVA* *0.7922*	*0.9973 (1 vs. 2)* *0.8323 (2 vs. 3)* *Sidak’s test*
	*2*	*UAS-Dα1-RNAi*	*R58E02-GAL4*	*8*	*0.4521*			
	*3*	*UAS-Dα1-RNAi*	*w*	*10*	*0.3557*			
Fig 4D	*1*	*w*	*R58E02-GAL4*	*8*	*0.3922*	*0.2810*	*One-way ANOVA* 0.0026 **	0.0038 (1 vs. 2) ** 0.0047 (2 vs. 3) ** *Sidak’s test*
	*2*	*UAS-Dα1-RNAi*	*R58E02-GAL4*	*7*	*NA*			
	*3*	*UAS-Dα1-RNAi*	*w*	*8*	*0.4805*			
Fig 4E	*1 (100:0)*	*w*	*R58E02-GAL4*	*12*	*NA*	*NA*	*Two-way ANOVA* 0.0447 (interaction) * < 0.0001 (mixture ratio) *** *0.5963 (genotype)*	*0.2725 (1 vs. 4)* *0.1378 (4 vs. 7)* *0.9517 (2 vs. 5)* *0.8016 (5 vs. 8)* *0.2275 (3 vs. 6)* 0.021 (6 vs. 9) * *Dunnett’s test*
	*2 (50:50)*	*w*	*R58E02-GAL4*	*12*	*NA*			
	*3 (0:100)*	*w*	*R58E02-GAL4*	*11*	*NA*			
	*4 (100:0)*	*UAS-Dα1-RNAi*	*R58E02-GAL4*	*12*	*NA*			
	*5 (50:50)*	*UAS-Dα1-RNAi*	*R58E02-GAL4*	*12*	*NA*			
	*6 (0:100)*	*UAS-Dα1-RNAi*	*R58E02-GAL4*	*10*	*NA*			
	*7 (100:0)*	*UAS-Dα1-RNAi*	*w*	*11*	*NA*			
	*8 (50:50)*	*UAS-Dα1-RNAi*	*w*	*10*	*NA*			
	*9 (0:100)*	*UAS-Dα1-RNAi*	*w*	*10*	*NA*			
S1 Fig	*1*	*tubP-GAL80ts, R57C10-GAL4*	*w*	*11*	*0.2464*	*NA*	*NA*	0.0127 (1 vs. 2) * *Mann Whitney test*
	*2*	*tubP-GAL80ts, R57C10-GAL4*	*UAS-Dα1-RNAi*	*10*	0.0012 **			
S2A Fig	*1*	*w*	*R58E02-GAL4*	*16*	*0.5674*	*0.9270*	*One-way ANOVA* 0.0018 **	0.0116 (1 vs. 2) * 0.0011 (2 vs. 3) ** *Holm-Sidak’s test*
	*2*	*UAS-Dα1-RNAi*	*R58E02-GAL4*	*16*	*0.4862*			
	*3*	*UAS-Dα1-RNAi*	*w*	*16*	*0.1013*			
S2B Fig	*1*	*w*	*R58E02-GAL4*	*16*	*0.5655*	*0.8303*	*One-way ANOVA* 0.0038 **	0.0065 (1 vs. 2) ** 0.0065 (2 vs. 3) ** *Holm-Sidak’s test*
	*2*	*UAS-Dα1-RNAi*	*R58E02-GAL4*	*16*	*0.2781*			
	*3*	*UAS-Dα1-RNAi*	*w*	*16*	*0.9405*			
S2C Fig	*1*	*w*	*R58E02-GAL4*	*23*	*0.1462*	*0.4454*	*One-way ANOVA* *0.8358*	*0.8004 (1 vs. 2)* *0.8097 (2 vs. 3)* *Holm-Sidak’s test*
	*2*	*UAS-Dα1-RNAi*	*R58E02-GAL4*	*24*	*0.8589*			
	*3*	*UAS-Dα1-RNAi*	*w*	*26*	*0.7899*			
S2D Fig	*1*	*w*	*R58E02-GAL4*	*23*	*0.6967*	*0.9752*	*One-way ANOVA* *0.1882*	*0.7002 (1 vs. 2)* *0.3211 (2 vs. 3)* *Holm-Sidak’s test*
	*2*	*UAS-Dα1-RNAi*	*R58E02-GAL4*	*24*	*0.8222*			
	*3*	*UAS-Dα1-RNAi*	*w*	*26*	*0.9293*			
S2E Fig	*1*	*w*	*R58E02-GAL4*	*8*	*0.3360*	*0.3646*	*One-way ANOVA* *0.3667*	*0.3371 (1 vs. 2)* *0.3371 (2 vs. 3)* *Holm-Sidak’s test*
	*2*	*UAS-Dα1-RNAi*	*R58E02-GAL4*	*8*	*0.1881*			
	*3*	*UAS-Dα1-RNAi*	*w*	*8*	*0.3757*			
S2F Fig	*1*	*w*	*R58E02-GAL4*	*8*	*0.1475*	*NA*	*Kruskal-Wallis test* *0.2751*	*0.4060 (1 vs. 2)* *> 0.9999 (2 vs. 3)* *Dunn’s test*
	*2*	*UAS-Dα1-RNAi*	*R58E02-GAL4*	*8*	0.0342 *			
	*3*	*UAS-Dα1-RNAi*	*w*	*8*	*0.5241*			
S2G Fig	*1*	*w*	*R58E02-GAL4*	*16*	*0.8082*	*0.6080*	*One-way ANOVA 0.2058*	*0.3058 (1 vs. 2)* *0.1800 (2 vs. 3)* *Sidak’s test*
	*2*	*UAS-Dα1-RNAi*	*R58E02-GAL4*	*14*	*0.6249*			
	*3*	*UAS-Dα1-RNAi*	*w*	*15*	*0.9485*			
S2H Fig	*1*	*R58E02-GAL4, UAS-Cas9.P2*	*+*	*8*	*0.7143*	*0.6359*	*One-way ANOVA* *0.2287*	*0.1936 (1 vs. 2)* *0.2135 (2 vs. 3)* *Holm-Sidak’s test*
	*2*	*R58E02-GAL4, UAS-Cas9.P2*	*sgRNA.Dα1*	*8*	*0.4987*			
	*3*	*+*	*sgRNA.Dα1*	*8*	*0.3516*			

### *Dα1* regulates learned odor choice

*Dα1*-mediated odor-evoked activity in PAM DANs suggested that *Dα1* may contribute to behavioral performance in odor-guided test conditions, rather than to sugar processing per se. We tested this by measuring appetitive memory in *Dα1* knockdown flies using assays that separate binary odor choice from single-odor pursuit. As expected, knockdown flies performed poorly in the standard two-odor choice task (Fig 4A). Separate analysis of the reciprocal PI1 and PI2 values showed reduced performance in both odor assignments, indicating that this phenotype was not specific to one odor condition (S2A, S2B Fig). However, when memory was assessed with a single conditioned odor against air, *Dα1* knockdown did not significantly reduce no-choice performance for either 4MCH or 3-OCT (Fig 4B, 4C). Here, ‘no-choice’ denotes testing a single odor against the odorless solvent control, rather than choosing between two odors. Consistent with this result, separate component plots of the same no-choice data showed that *Dα1* knockdown did not significantly alter responses to either the sucrose-paired odor or the unpaired odor when each was tested against air (S2C, S2D Fig). Effect sizes and confidence intervals for these main no-choice comparisons are reported in S1 Table. This result was further supported by no-choice tests using lower odor concentrations, in which *Dα1* knockdown also did not significantly reduce performance (S2E, S2F Fig). Knockdown flies also performed worse when choosing between the conditioned odor and a novel odor (Fig 4D), whereas choices based on innate odor valence were unaffected (S2G Fig). Similarly, *Dα1* knockout did not significantly reduce no-choice performance at a lower odor concentration (S2H Fig).

**Fig 4:**
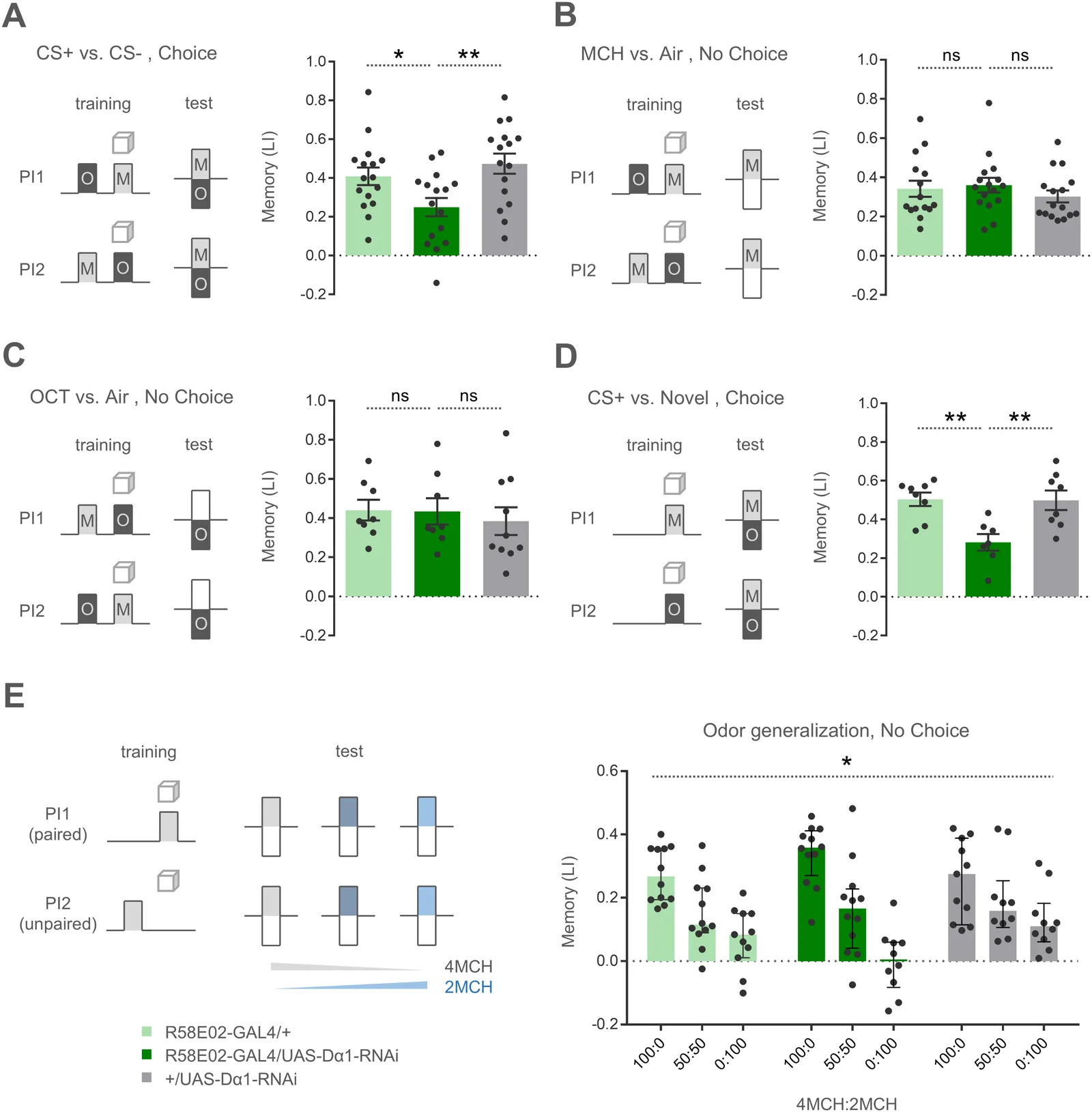
Dα1 in the PAM DANs mediates learned odor choice. **(A)** Dα1 knockdown in PAM neurons impaired appetitive memory in a forced two-odor discrimination task. *p < 0.05, **p < 0.01, Sidak’s multiple comparisons test; N = 16. **(B, C)** Single-odor no-choice tests in which the same test odor was evaluated after reciprocal paired or unpaired training. The response to 4MCH versus air was tested after 4MCH had been experienced either as sucrose-paired or sucrose-unpaired **(B)**. The same design was used for 3-OCT **(C)**. Dα1 knockdown did not significantly reduce learned odor pursuit for 4MCH (B; Dunn’s multiple comparisons test; N = 15, 16, and 16) or 3-OCT (C; Sidak’s multiple comparisons test; N = 8, 8, and 10). **(D)** Dα1 knockdown impaired choice between the conditioned odor and a novel odor. **p < 0.01, Sidak’s multiple comparisons test; N = 8, 7, and 8. **(E)** Odor generalization assay using mixtures of the trained odor 4MCH and the similar odor 2MCH. Flies were tested with 4MCH/2MCH mixtures at the indicated ratios after paired or unpaired training with 4MCH. Dα1 knockdown did not increase generalization to similar odors. Individual points indicate biological replicates. Bars indicate mean ± SEM. Asterisks indicate significant genotype × mixture ratio interactions (*p < 0.05, two-way ANOVA, N = 12, 12, 11, 12, 12, 10, 11, 10, and 10). Complete statistical results are provided in Table 1.

One potential explanation is reduced odor specificity, leading to greater generalization between odors [48,72]. To test this, we conditioned flies with one odor and then probed memory using a second, perceptually similar odor (Fig 4E). *Dα1* knockdown flies did not show increased generalization. Instead, they generalized less than controls (Fig 4E) (F(4, 91) = 2.546, p = 0.0447 (interaction); F(2, 91) = 33.14, p < 0.0001 (mixture ratio); F(2, 91) = 0.5199, p = 0.5963 (genotype), two-way ANOVA), arguing against overgeneralization as the source of the phenotype. Together, these results indicate that *Dα1*-dependent signaling in PAM neurons supports normal learned odor-choice performance under conditions in which flies choose between competing odor alternatives.

## Discussion

We examined the role of nicotinic acetylcholine receptors (nAChRs) in reward-related PAM DANs. Among nine nAChR genes tested, selectively disrupting *Dα1* in PAM DANs impaired appetitive memory (Fig 1). *Dα1* was present in these neurons at both the transcript and protein levels (Fig 2). Live imaging showed that *Dα1* knockdown in PAM DANs attenuated odor-evoked synaptic responses, whereas sugar-evoked responses were largely preserved (Fig 3), indicating that *Dα1*-dependent nicotinic signaling preferentially supports odor-evoked PAM terminal activity. Consistent with this, *Dα1* downregulation reduced performance in a forced two-odor discrimination task (Fig 4). By contrast, we detected no significant reduction across several single-odor pursuit assays, including 4MCH- and 3-OCT-centered no-choice assays and additional lower-concentration no-choice assays (Figs 4B, 4C, S2E, S2F, S2H). This consistent pattern suggests that *Dα1*-dependent signaling is particularly important under competing-odor choice conditions, although differences in task demands and smaller effects on single-odor pursuit cannot be excluded. The preservation of sugar-evoked calcium responses (Fig 3) suggests that canonical appetitive reinforcement pathways [28,29,39,73] are largely functional. Together, these findings support a role for *Dα1*-dependent nicotinic signaling in odor-evoked PAM terminal activity and learned odor-choice performance, particularly when competing odor cues are present.

These findings are consistent with broader evidence that nAChRs modulate DAN activity, dopamine release, and dopamine-dependent plasticity. Mammalian studies have shown that nAChRs regulate midbrain DAN activity and can interact with dopamine-dependent mechanisms of striatal synaptic plasticity [74,75]. Our results extend this ACh-DA framework to *Drosophila* PAM DANs by showing that *Dα1*-dependent nicotinic signaling preferentially supports odor-evoked synaptic activity while leaving sugar-evoked responses largely preserved.

Because the PAM-directed *Dα1* manipulations were not temporally restricted, developmental contributions cannot be excluded. Nevertheless, the selective attenuation of odor-evoked responses in adult PAM terminals, together with preserved sugar responses and basic sensory behavior, supports a specific requirement for *Dα1* in normal mature PAM circuit function.

The preserved sugar-evoked responses also indicate that the mechanism mediating this *Dα1*-independent PAM activation remains unresolved. Odor and sugar stimulation may recruit distinct afferent pathways or transmitter systems in PAM neurons. Such *Dα1*-independent signaling could involve nAChRs containing other subunits, muscarinic acetylcholine receptors, or non-cholinergic reward-related inputs. Larval work showing short-neuropeptide-F-dependent KC-to-pPAM feedback provides one example that reward-related DAN modulation need not be exclusively cholinergic [76]. Future receptor- and pathway-specific perturbations will be needed to identify the mechanism mediating the preserved sugar response.

These findings raise the question of how *Dα1*-dependent odor-evoked PAM activity could be linked to learned odor-choice performance at the circuit level. The odor-selective requirement for *Dα1* is consistent with a cholinergic pathway that conveys odor-related information to PAM neurons. Because direct PN input to PAM neurons appears limited [54], odor-related cholinergic signals to PAM are likely to arise largely downstream of PNs. The MB provides a direct substrate: PNs deliver strong odor drive to cholinergic KCs in the MB calyx [30,51], sparse ensembles of which encode fine odor distinctions through population activity [23–27]. Ultrastructural datasets reveal extensive reciprocal connectivity between KCs and DANs across the MB lobes [31,32]: individual PAM DANs receive hundreds of KC-to-DAN synapses, in some cases exceeding the number of canonical DAN-to-KC contacts [32]. This architecture makes PAM axon terminals a likely locus for cholinergic modulation. Consistent with this, and with evidence that cholinergic control can operate at DAN axon terminals [4], we detected Dα1 puncta in PAM axon-terminal regions (Fig 2).

Our results indicate that *Dα1*-dependent signaling in PAM DANs is required for normal performance in learned odor-choice contexts with competing odor alternatives, although the underlying neural basis remains unclear. In the MB lobes, learned odor perception depends on odor-evoked activity patterns at KC synapses [77,78] and MBON synapses [79]. KCs and MBONs generate sparse, temporally structured representations [23,25,26] that retain enough information for odor discrimination [77,78] and categorization [25,80,81]. MB odor representations also remain dynamic, and recurrent MB feedback can reshape them [27,72], while dopaminergic signals modulate them across compartments through specific receptors [82]. Sparse KC ensembles may convey learned-odor information to PAM DANs through KC-to-DAN synapses, and reciprocal DAN-to-KC contacts could return dopaminergic signals to the same or neighboring KC synapses [4,31,32]. Within this loop, one possibility is that Dα1-dependent odor-evoked PAM activity influences how learned and competing odor representations are weighted during choice.

In parallel, and not mutually exclusively, MBON-guided indirect pathways may also shape odor choice. Consistent with the behavioral dissociation observed here, distinct combinations of MBONs support memory-guided odor choice and odor-source attraction [83]. In flies, the PAM-β′2a population (Fig 2) contributes to the integration of conflicting sensory cues [42], encodes punishment omission during reversal learning [84], and can signal relative “better-than” value during sequential aversive experiences [85]. One of its postsynaptic partners, MBON-γ5β′2a, undergoes learning-related plasticity during extinction and reversal learning, contributing to interactions between original and newly formed memories [30,84,86]. Moreover, the cholinergic MBON-γ2α′1 [35], which encodes transitions between two odors experienced in sequence [81], provides direct excitatory input to PAM-β′2a [35,84]. Accordingly, Dα1 puncta in putative dendritic regions (Fig 2J, 2K) may participate in local computations within this cholinergic feedback loop [35,84]. Because PAM-β′2a can encode relative value [85], such cholinergic input could influence value-comparison signals, although this possibility remains to be tested. In macaques, anterior insular activity reflects reference-dependent decision variables [87], whereas parietal value signals scale with the set of available alternatives [88]. Parietal neurons also integrate reward history to encode the relative value of competing options [89]. This raises the possibility that analogous integrative computations within MB circuitry support relative-value coding between learned and reference odors and thereby influence choice decisions in flies.

Together, these circuit considerations suggest possible routes by which *Dα1*-dependent cholinergic signaling in PAM neurons could influence learned odor choice. The present study identifies *Dα1*-dependent odor-evoked PAM terminal activity as a cellular entry point into this problem, but the downstream mechanism remains to be defined. Future experiments measuring dopamine release, conditioning-dependent changes in odor-evoked PAM activity, PAM responses during simultaneous presentation of competing odor cues, and odor–sugar integration will be needed to determine how this activity is transformed into dopamine output and choice-related behavior.

## Materials and methods

### Flies

Canton-S was used as a wild-type strain. *w;;R58E02-GAL4, w;;R15A04-GAL4* [90], *w;nAChRα1-2A-GAL4* [67], *yw; nAChRα1*^*MI00453-GFSTF.0*^ (BDSC #60150) [69], *MB247-GAL80* (BDSC #64306), *w;;UAS-Dα1-RNAi* (BDSC #28688), *w;UAS-Dα2-RNAi* (BDSC #27493), *w;UAS-Dα3-RNAi* (BDSC #61225), *w;UAS-Dα4-RNAi* (BDSC #31985), *w;UAS-Dα5-RNAi* (BDSC #77418), *w;UAS-Dα6-RNAi* (BDSC #57818), *w;UAS-Dα7-RNAi* (BDSC #51049), *w;UAS-Dβ1-RNAi* (BDSC #31883), *w;UAS-Dβ3-RNAi* (BDSC #25927) [91], *w;;UAS-mCD8::GFP* (BDSC #32194) [92], *w; UAS-GCaMP6s.brpS.mCherry* (BDSC #77131) [71], *UAS-KDR.PEST* (BDSC #55791), *Dα1*^*KDRT-STOP-smOLLAS*^ (BDSC #602271) [70], *UAS-Cas9.P2* (BDSC #58985), *sgRNA.Dα1* (BDSC #79346), *w, R58E02-p65.AD* (BDSC #601788), *w;; UAS-LexA.DBD* (BDSC #56529), *w;; LexAop2-myr::GFP* (BDSC #32209), *w;tubP-GAL80ts* (BDSC #7019)*, w;;R57C10-GAL4* (BDSC #39171), *w; UAS-mCD8::RFP* (a gift from Dr Ilona Kadow) were previously described. Flies were raised at 24°C with 12:12 LD cycle. Knock-down flies were prepared as the F1 progeny of the crosses between females of *w;;R58E02-GAL4* or *w* and males of UAS effectors or CS. The F1 progeny was raised at 24°C, aged 3–12 d after eclosion before experiments. To visualize *Dα1* transcriptional reporter expression, females of *w;;UAS-mCD8::GFP* were crossed to males of *w; nAChRα1-2A-GAL4* (Fig 2A-2C). For intersectional labeling, males of *w, R58E02-p65.AD;; UAS-LexA.DBD, LexAop2-myr::GFP* were crossed to females of *w; nAChRα1-2A-GAL4* (Fig 2D-2F). For PAM-restricted Dα1-smOLLAS labeling, females of *w;;R58E02-GAL4, UAS-mCD8::RFP* were crossed to males of *w; UAS-KDR.PEST*; *Dα1*^*KDRT-STOP-smOLLAS*^ (Fig 2J-2L). The F1 flies used for whole-mount immunohistochemistry were aged 3–10 d after eclosion. For live imaging experiments, males of *w;;R15A04-GAL4* were crossed to *w;UAS-GCaMP6s.brpS.mCherry* or *w;UAS-GCaMP6s.brpS.mCherry;UAS-Dα1-RNAi* females. The F1 flies were raised at 24°C, aged to 3–8 d after eclosion before experiments (Fig 3). A complete list of the fly crosses for experiments is listed in Table 1.

### Behavioral assays

The conditioning and testing protocols were as previously described [38,39,93]. In brief, a group of approximately 50 flies was placed in a training tube where they alternately received octan-3-ol (3-OCT; Merck) and 4-methylcyclohexanol (4MCH; Sigma-Aldrich) for 1 minute in a constant air stream, with or without dried 2M sucrose paper (Figs 1, 4, S2). Training stimulus presentations were separated by a 1-min interstimulus interval (ISI), including odor-to-odor, odor-to-sucrose, and sucrose-to-odor intervals in the paired and unpaired protocols. Immediately after training, the flies were given a choice between sugar-paired (CS+) and unpaired (CS-) odors for 2 minutes in a T maze. In the single-odor no-choice protocols (Fig 4B, 4C), the same test odor was measured after reciprocal training in which that odor had been experienced either as the sucrose-paired odor (CS+) or the unpaired odor (CS−). Thus, in the 4MCH-centered no-choice assay, both reciprocally trained groups were tested for preference to 4MCH versus paraffin oil; in the 3-OCT-centered no-choice assay, both reciprocally trained groups were tested for preference to 3-OCT versus paraffin oil. This design kept test-odor identity constant across reciprocal conditions. For the novel odor protocol (Fig 4D), flies were trained using either 4MCH and paraffin oil or 3-OCT and paraffin oil for 1 minute in a constant air stream with or without dried 2M sucrose paper. After training, the flies were given a choice between CS+ and novel odors for 2 minutes in a T maze. In the innate choice protocol (S2G Fig), flies were mock-trained without any odor or sugar and then given a choice between two odors for 2 minutes in a T maze. For the odor generalization protocol (Fig 4E), flies were trained with 4MCH using paired and unpaired sucrose protocols. In the paired condition, 4MCH was presented together with dried 2 M sucrose paper, whereas in the unpaired condition, 4MCH and sucrose were presented separately with the 1-min ISI described above. Immediately after training, flies were tested with mixtures of 4MCH and the similar odor 2MCH at the indicated ratios. This assay was used to test whether *Dα1* knockdown altered generalization from the trained odor to a similar odor. Odors were diluted to 10% in paraffin oil and placed in cups with either 3 mm (OCT) or 5 mm (MCH) diameters. For each test, a preference index (PI) was calculated as the number of flies choosing one option minus the number choosing the alternative option, divided by the total number of flies tested. In two-odor choice assays, the two options were the sucrose-paired and unpaired odors. In single-odor no-choice assays, the two options were the test odor and paraffin oil. For reciprocal conditioning experiments, two preference indices were calculated from the two reciprocal training groups. The learning index (LI) was then calculated as (PI1 − PI2)/2, with the sign arranged so that positive LI values indicate learned preference for the sucrose-paired odor. In the sugar preference test (Fig 1F), flies were given a choice between dried 2M sucrose paper and control paper for 2 minutes in a T maze. For the odor avoidance test (Fig 1D, 1E), flies were given a choice between 4MCH and paraffin oil, or 3-OCT and paraffin oil for 2 minutes in a constant air stream.

### RT–qPCR

RT-qPCR was performed as previously described [94] with minor modifications. For adult-specific pan-neuronal knockdown, *UAS-Dα1-RNAi* was expressed using *tubP-GAL80ts; R57C10-GAL4*. Flies were reared at 18°C and shifted to 29°C for 3 days as adults before head collection. Driver-only flies subjected to the same temperature regimen served as controls. Frozen adult heads were homogenized in 50 uL ice-cold lysis buffer containing 20 mM Tris-HCl (pH 7.5), 150 mM NaCl, 5 mM MgCl_2_, 1 mM DTT, and 1% Triton X-100 using a Multi-Beads Shocker (Yasui Kikai) at 3,000 rpm for 15 s. The lysate was thawed at 4°C, centrifuged, and RNA from the supernatant was purified by TRIzol LS Reagent (10296010, Invitrogen) and the Direct-zol RNA MicroPrep kit (R2062; Zymo Research) according to the manufacturer’s instructions. RNA concentrations were measured using a NanoDrop spectrophotometer and adjusted to comparable concentrations among samples. cDNA was synthesized using ReverTra Ace qPCR RT Master Mix with gDNA Remover (FSQ-301; Toyobo) and qPCR was performed using TB Green Premix Ex Taq II (RR830A; Takara Bio) on CFX Connect Real-Time PCR Detection System (Bio-Rad). Relative *Dα1* expression was calculated by dividing 2^−Cq(*Dα1*) by 2^−mean Cq(*Rp49*, *αTub84B*) for each biological replicate. The resulting values were then normalized to the mean of the driver-only control group. The primer sequences (5′–3′) were as follows: *Dα1*, forward, TCTCACCGTCAAGATGGGTCT, and reverse, TCGTTCCATTCCTGTTCCACC; *Rp49*, forward, AGCATACAGGCCCAAGATCG, and reverse, TGTTGTCGATACCCTTGGGC; and *α-Tub84B*, forward, GATCGTGTCCTCGATTACCGC, and reverse, GGGAAGTGAATACGTGGGTAGG.

### Brain dissection and Immunohistochemistry

The dissection of fly brains was carried out as previously described [67], with minor modifications. Female fly brains were dissected in phosphate-buffered saline (PBS) and pre-fixed in 1% paraformaldehyde (PFA) in PBS on ice for up to 30 minutes. They were then fixed in 2% PFA in PBS for one hour at room temperature. After fixation, the brains were washed in PBT (0.1% Triton X-100 in PBS) three times for 10 minutes each. Immunostaining was performed as previously described [67]. The following primary antibodies were employed at the specified dilutions: rabbit anti-GFP (1:1000; Invitrogen; A11122), mouse anti-TH (1:100; ImmunoStar Inc.; 22941) and rabbit anti-smOLLAS (1:20–100; Osenses; OSL00028G). For the secondary antibodies, the following were used at the indicated dilutions: AlexaFluor-488 goat anti-rabbit (1:1000; Invitrogen; A11034) and AlexaFluor-568 goat anti-mouse (1:1000; Invitrogen; A11004). An 86% glycerol solution or SeeDB2 [95] was used as the mounting medium, and either native or immunostained fluorescence images were captured.

### Confocal imaging for histology

Imaging was performed on the Olympus FV1200 confocal microscope with GaAsP sensors. A 30x/1.05 silicone immersion objective (UPLSAPO30XS, Olympus) or a 60x/1.42 oil immersion objective (PLAPON60XO, Olympus) (Fig 2) was used for scanning specific regions of interest. For Fig 2D and 2E, Zeiss LSM780 confocal microscope with GaAsP sensors and a 63x/1.40 oil immersion objective (Plan-Apochromat 63x, Zeiss) was used. Confocal stacks were analyzed with the open-source software Image-J (National Institutes of Health) and Fiji [96]. Where appropriate, 2D/3D image deconvolution was applied using Diffraction PSF 3D and Parallel Iterative Deconvolution plugins in Image-J.

### *In vivo* live imaging

Flies were prepared as previously described [48] with some modifications. The fly was briefly anesthetized on ice and placed in a custom-made holding device on a Peltier plate (CP-085, Scinics) held at 4 °C. The head capsule was fixed to the dish by UV curing optical adhesives (NOA68, Thorlabs). The proboscis was glued onto the capsule to eliminate brain movement. Forelegs interfering sugar feeding during recordings were removed. A small window on the top of the head capsule was opened using sharp forceps in *Drosophila* saline (103 mM NaCl (31320-05, Nacalai tesque), 3 mM KCl (28514-75, Nacalai tesque), 5 mM TES (32810-55, Nacalai tesque), 8 mM Trehalose (Tokyo kasei kogyo), 10 mM D-glucose (16806-25, Nacalai tesque), 26 mM NaHCO3 (31213-15, Nacalai tesque), 1 mM NaH2PO4 (A0110846 010, Merck), 1.5 mM CaCl2 (C5080, Sigma-Aldrich), 4 mM MgCl2 (M2670, Sigma-Aldrich), ~ 270 mOsm/kg, pH ~ 7.2). Air sacs and fat bodies covering the brain surface were carefully removed.

A laser-scanning confocal microscope (A1R, Nikon) equipped with a 25x/1.1 water immersion objective (Apo LWD 25x, Nikon) and a Piezo nanopositioner (Nano-F450, MCL Inc.) was utilized for live imaging. GCaMP6s and mCherry were sequentially excited at 488 nm and 561 nm, respectively. Emission light was collected by GaAsP detectors using dichroic mirrors and emission filters (BP500–550 and BP570-620). Transverse sections of the mushroom body lobes and the superior medial protocerebrum were scanned at a resolution of 0.33 μm per pixel (512 × 128 pixels), capturing images at a rate of 1 frame per second with line scans averaged 16 times in resonant scanning mode. The pinhole was set to 2.5 A.U. (for 561 nm). Images were acquired over a duration of 20 or 30 seconds and saved for later processing.

For sugar stimulation, a droplet of 500 mM sucrose was applied to the tip of a Microloader pipette (Eppendorf) and presented to the glued proboscis for 3 seconds using a micromanipulator (UN-3C, Narishige). The sugar stimulation of the flies was monitored with a USB camera (Grasshopper3, FLIR) attached to a zoom lens (MACRO ZOOM 0.3 × -1 × 1:4.5, Computar) and recorded using FlyCapture2 (FLIR). Odor stimulation was conducted automatically using a custom-made olfactometer. By controlling a solenoid valve through the confocal system, scented air (approximately 50 mL/min) with 10% paraffin-diluted 4MCH or 3-OCT was injected into a constant air stream (approximately 450 mL/min) without altering the total flow (around 500 mL/min). During each stimulation, approximately 25 mL of odor-containing air was delivered to a fly over a 3-second period through a 4 mm silicone tube positioned about 10 mm away from the fly’s head.

### Connectome analysis of cholinergic inputs to PAM neurons

Connectome analyses were performed using the adult *Drosophila* hemibrain connectome dataset (hemibrain:v1.2.1) accessed through neuPrint and neuprint-python [55,97]. PAM dopamine neurons were identified from neuPrint annotations as neurons whose type field started with “PAM.” We focused on PAM neuron classes corresponding to the γ5, β′2, β′1, and α1 regions analyzed in this study. Synapse-level connections from presynaptic neurons to these PAM neurons were retrieved using fetch_synapse_connections with SynapseCriteria (primary_only = True). To localize inputs onto PAM neurons, synapses were classified according to the postsynaptic PAM-side ROI (roi_post). MB-lobe synapses were defined as postsynaptic PAM sites in gL, bL, b′L, aL, or a′L ROIs. MB-lobe-external synapses were defined as synapses whose postsynaptic PAM-side ROI was outside these MB lobe ROIs, and were subdivided into CRE, SMP, and other external regions. KC-to-PAM synapses within MB lobe regions were summarized separately from predicted or curated cholinergic inputs outside the MB lobes.

Cholinergic presynaptic neurons were defined using published neurotransmitter predictions and curated cell-type information. Neuron-level neurotransmitter predictions from Eckstein et al. were used to identify predicted cholinergic neurons, using acetylcholine as the predicted transmitter with confidence probability ≥ 0.7 [98]. Kenyon cells were treated as cholinergic based on established cell identity. MBON transmitter identities were curated from published assignments when available, with known non-cholinergic MBON identities prioritized over neuron-level predictions [31,35]. Synapse counts indicate individual synaptic connections. Input-neuron counts indicate unique presynaptic bodyIds within each category, and target-PAM counts indicate unique postsynaptic PAM bodyIds. Because the same presynaptic neuron can contribute synapses in more than one external ROI, regional input-neuron counts do not necessarily sum to the total input-neuron count.

### Data analyses

All acquired images were initially processed using Fiji. To stabilize an object in each recording, the TurboReg plugin [99] was applied using the mCherry signal. The GCaMP6s signal was normalized by dividing it by the mCherry signal (GCaMP6s/mCherry) to account for differences in sensor abundance. Regions of interest (ROIs) for subsequent calculations were defined based on the mCherry signal in both the left and right hemispheres. Pixels without a mCherry fluorescence value were excluded from analysis. After this, the image stacks were imported into MATLAB (MathWorks) and log-transformed for calculations. For Fig 3, imaging responses were summarized as mean log_10_(GCaMP6s/mCherry) values during pre-stimulus, stimulus, and post-stimulus epochs.

### Statistical analyses

Statistics were performed by Prism6 (Graphpad). For the data points that did not violate the assumption of normality and homogeneity of variance (D’Agostino & Brown-Forsythe test), parametric statistics were applied. The data points that were significantly different from the normal distribution were analyzed with nonparametric statistics. Based on the standard practice in the field, alpha was set to 0.05. For Fig 3C-3E, genotype effects across epochs were analyzed using two-way repeated-measures ANOVA, with genotype as the between-subject factor and epoch as the within-subject factor. For the main no-choice comparisons shown in Fig 4B and 4C, *Dα1*-KD flies were compared with each genetic control separately. Mean differences were calculated as *Dα1*-KD minus control performance-index values. Effect sizes are reported as mean differences in performance-index units and as Cohen’s d, with confidence intervals for the mean differences provided in S1 Table. The complete experimental design and statistical results are described in Table 1.

## Supporting information

S1 Fig qRT-PCR validation of the Dα1 RNAi reagent. Adult-specific pan-neuronal expression of UAS-Dα1-RNAi was induced using tubP-GAL80ts; R57C10-GAL4. Flies were reared at 18°C, and adults were shifted to 29°C for 3 days before head collection. Dα1 transcript levels were quantified by qRT-PCR and normalized to the reference genes Rp49 and αTub84B. Individual points represent biological replicates (N = 11 and 10 for the control and Dα1-RNAi groups, respectively), and bars indicate mean ± SD. *p < 0.05. Complete statistical results are provided in Table 1.
(PDF)

S2 Fig Additional behavioral controls for Dα1-dependent learned odor choice. (**A-B**) PI1 and PI2 values for the reciprocal two-odor discrimination assay shown in Fig 4A. Dα1 knockdown reduced performance in both reciprocal odor assignments. Preference to 4MCH (A; Holm-Sidak’s multiple comparisons test; N = 16) and preference to 3-OCT (B; Holm-Sidak’s multiple comparisons test; N = 16) are shown. (**C, D**) Component preference indices underlying the single-odor no-choice learning indices shown in Fig 4B and 4C. The same data were re-plotted according to training contingency, showing preference to the sucrose-paired odor (CS+) versus air (C; Holm-Sidak’s multiple comparisons test; N = 23, 24, and 26) and preference to the unpaired odor (CS−) versus air (D; Holm-Sidak’s multiple comparisons test; N = 23, 24, and 26). (**E, F**) Single-odor no-choice tests using lower concentrations of 4MCH at 1:100 (E; Holm-Sidak’s multiple comparisons test; N = 8) and 1:400 (F; Dunn’s multiple comparisons test; N = 8). (**G**) Innate odor choice between 4MCH and 3-OCT. Individual points indicate biological replicates. Sidak’s multiple comparisons test; N = 16, 14, and 15. (**H**) Single-odor no-choice tests (1:100 4MCH) with PAM-specific conditional knockout of Dα1 using R58E02-GAL4-driven Cas9 and Dα1 sgRNA. Holm-Sidak’s multiple comparisons test; N = 8. Bars indicate mean ± SEM. ns, not significant; *p < 0.05, **p < 0.01. Complete statistical results are provided in Table 1.
(PDF)

S1 Table Statistical summary of the main no-choice comparisons shown in Fig 4B and 4C. Mean differences were calculated as Dα1-KD minus the indicated genetic control; negative values indicate lower performance in Dα1-KD flies. Effect sizes are reported as mean differences in performance-index units and as Cohen’s d. Confidence intervals indicate the uncertainty of the mean difference. P values are from Welch’s t-tests.
(PDF)

S2 Table Connectome-based summary of predicted and curated cholinergic inputs to PAM neuron classes analyzed in this study. Synapse-level inputs were summarized from the adult *Drosophila* hemibrain connectome for PAM neuron classes corresponding to the γ5, β′2, β′1, and α1 regions analyzed in this study. PAM type names and compartment annotations follow neuPrint hemibrain annotations. PAM03 is shown separately as a β2/β′2a mixed PAM type. MB-lobe inputs indicate KC-to-PAM synapses within MB lobe regions. Outside-MB inputs indicate predicted or curated cholinergic inputs to PAM-side postsynaptic sites outside MB lobe regions, subdivided into CRE, SMP, and other external regions. Synapse counts indicate individual synaptic connections. Input-neuron counts indicate unique presynaptic bodyIds within each category. Because the same presynaptic neuron can contribute synapses in more than one external region, regional input-neuron counts do not necessarily sum to the total input-neuron count. Target PAM neurons indicate unique postsynaptic PAM bodyIds.
(PDF)

S1 Data Numerical data underlying Figs 1A–1F, 3C–3E, 4A–4E, S1, and S2A – S2H. Data are organized in separate worksheets labeled by figure panel and, where applicable, stimulus condition.
(XLSX)
