# Supplementary material for: Nicotinic receptor in dopamine neurons mediates learned odor choice
Source: PLoS Genet. 2026 Oct 29;22(10):e1012356. doi: 10.1371/journal.pgen.1012356 (PMC13739223; doi:10.1371/journal.pgen.1012356)
Supplement: Supplementary file 1 [file pgen.1012356.s001.pdf]

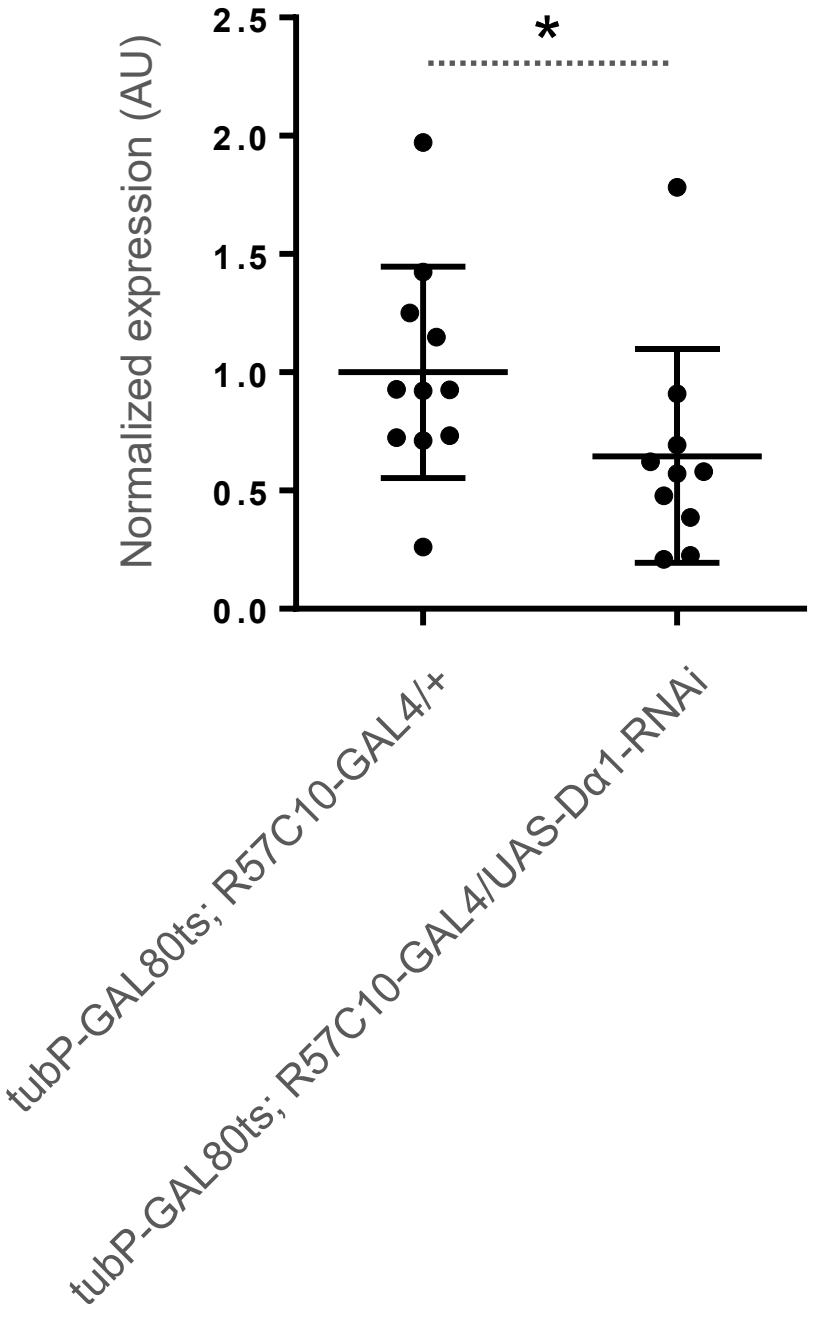

Normalized expression (AU)
2.5
2.0
1.5
1.0
0.5
0.0
*
tubP-GAL80ts; R57C10-GAL4/+
tubP-GAL80ts; R57C10-GAL4/UAS-Dα1-RNAi
