# Supplementary material for: Nicotinic receptor in dopamine neurons mediates learned odor choice
Source: PLoS Genet. 2026 Oct 29;22(10):e1012356. doi: 10.1371/journal.pgen.1012356 (PMC13739223; doi:10.1371/journal.pgen.1012356)
Supplement: Supplementary file 2 [file pgen.1012356.s002.pdf]

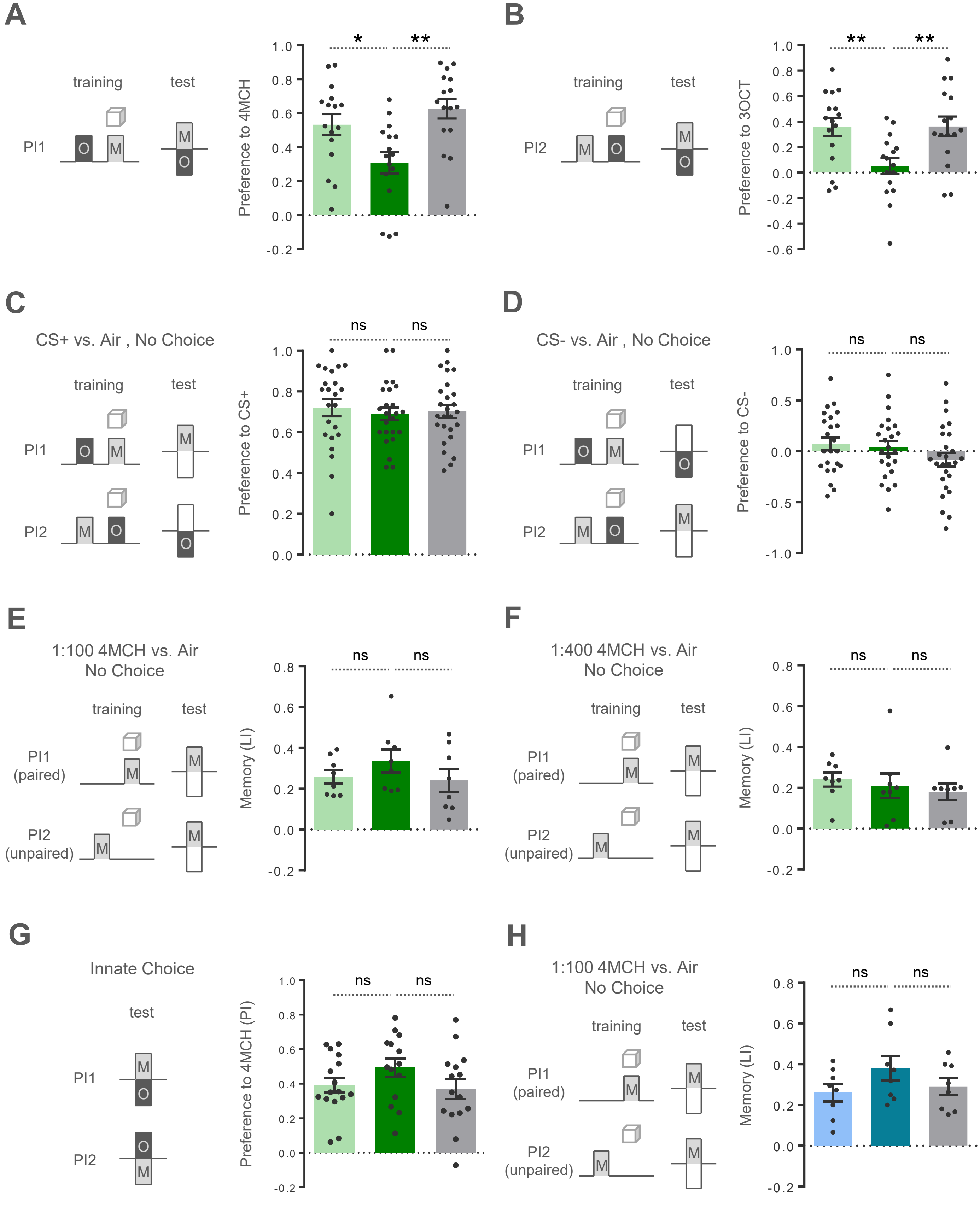

R58E02-GAL4/+
R58E02-GAL4/UAS-Dα1-RNAi
+/UAS-Dα1-RNAi

R58E02-GAL4/UAS-Cas9.P2
R58E02-GAL4/UAS-Cas9.P2, sgRNA.Dα1
+/sgRNA.Dα1
