# Supplementary material for: Nicotinic receptor in dopamine neurons mediates learned odor choice
Source: PLoS Genet. 2026 Oct 29;22(10):e1012356. doi: 10.1371/journal.pgen.1012356 (PMC13739223; doi:10.1371/journal.pgen.1012356)
Supplement: Supplementary file 3 [file pgen.1012356.s003.pdf]

Table S1. Effect sizes and confidence intervals for single-odor no-choice performance.

Assay	Comparison	Mean difference	95% CI	Cohen’s d	Welch p
MCH vs air (Fig 4B)	KD vs GAL4 control	0.018	−0.095 to +0.132	0.118	0.746
MCH vs air (Fig 4B)	KD vs UAS-RNAi control	0.057	−0.041 to +0.156	0.422	0.242
OCT vs air (Fig 4C)	KD vs GAL4 control	−0.006	−0.190 to +0.178	−0.037	0.942
OCT vs air (Fig 4C)	KD vs UAS-RNAi control	0.05	−0.157 to +0.257	0.239	0.615
