# Supplementary material for: Nicotinic receptor in dopamine neurons mediates learned odor choice
Source: PLoS Genet. 2026 Oct 29;22(10):e1012356. doi: 10.1371/journal.pgen.1012356 (PMC13739223; doi:10.1371/journal.pgen.1012356)
Supplement: Supplementary file 4 [file pgen.1012356.s004.pdf]

Table S2. Connectome-based summary of predicted and curated cholinergic inputs to PAM neuron classes analyzed in this study.

Anatomical group	PAM type	PAM instance / compartment annotation	KC→PAM synapses in MB lobes	KC input neurons	Target PAM neurons in MB lobes
γ5	PAM01_a	PAM01_a(y5)_L; PAM01_a(y5)_R	11258	858	25
γ5	PAM01_b	PAM01_b(y5)_L; PAM01_b(y5)_R	4068	778	14
γ5/β′2a mixed	PAM15_a	PAM15_a(y5B'2a)_L; PAM15_a(y5B'2a)_R	771	343	2
γ5/β′2a mixed	PAM15_b	PAM15_b(y5B'2a)_L; PAM15_b(y5B'2a)_R	1696	616	3
β′2-associated	PAM06_a	PAM06_a(B'2m)_L; PAM06_a(B'2m)_R	7926	370	24
β′2-associated	PAM06_b	PAM06_b(B'2m)_L; PAM06_b(B'2m)_R	2996	408	9
β′2-associated	PAM02	PAM02(B'2a)_L; PAM02(B'2a)_R	6604	541	15
β′2-associated	PAM05	PAM05(B'2p)_L; PAM05(B'2p)_R	5531	419	18
β2/β′2a mixed	PAM03	PAM03(B2B'2a)_L; PAM03(B2B'2a)_R	865	556	7
β′1	PAM13	PAM13(B'1ap)_L; PAM13(B'1ap)_R	2413	258	14
β′1	PAM14	PAM14(B'1m)_L; PAM14(B'1m)_R	2753	296	16
α1	PAM11	PAM11(a1)_L; PAM11(a1)_R	6240	920	14

Anatomica l group	PAM type	PAM instance / compartment annotation	Outside-MB predicted cholinergic synapses: total	CRE synapses	SMP synapses	Other external ROI synapses
γ5	PAM01_a	PAM01_a(y5)_L; PAM01_a(y5)_R	812	37	679	96
γ5	PAM01_b	PAM01_b(y5)_L; PAM01_b(y5)_R	352	41	300	11
γ5/β′2a mixed	PAM15_a	PAM15_a(y5B'2a)_L; PAM15_a(y5B'2a)_R	101	3	84	14
γ5/β′2a mixed	PAM15_b	PAM15_b(y5B'2a)_L; PAM15_b(y5B'2a)_R	82	19	63	0
β′2-associated	PAM06_a	PAM06_a(B'2m)_L; PAM06_a(B'2m)_R	1868	791	828	249
β′2-associated	PAM06_b	PAM06_b(B'2m)_L; PAM06_b(B'2m)_R	216	66	127	23
β′2-associated	PAM02	PAM02(B'2a)_L; PAM02(B'2a)_R	1052	87	850	115
β′2-associated	PAM05	PAM05(B'2p)_L; PAM05(B'2p)_R	1266	525	639	102
β2/β′2a mixed	PAM03	PAM03(B2B'2a)_L; PAM03(B2B'2a)_R	111	45	51	15
β′1	PAM13	PAM13(B'1ap)_L; PAM13(B'1ap)_R	843	441	341	61
β′1	PAM14	PAM14(B'1m)_L; PAM14(B'1m)_R	851	342	263	246
α1	PAM11	PAM11(a1)_L; PAM11(a1)_R	1002	12	228	762

**Anatomical group**	**PAM type**	**PAM instance / compartment annotation**	**Outside-MB predicted cholinergic input neurons: total**	**CRE input neurons**	**SMP input neurons**	**Other external input neurons**
γ5	PAM01_a	PAM01_a(y5)_L; PAM01_a(y5)_R	152	18	111	47
γ5	PAM01_b	PAM01_b(y5)_L; PAM01_b(y5)_R	85	12	75	9
γ5/β′2a mixed	PAM15_a	PAM15_a(y5B'2a)_L; PAM15_a(y5B'2a)_R	36	3	26	10
γ5/β′2a mixed	PAM15_b	PAM15_b(y5B'2a)_L; PAM15_b(y5B'2a)_R	44	13	35	0
β′2-associated	PAM06_a	PAM06_a(B'2m)_L; PAM06_a(B'2m)_R	215	134	115	64
β′2-associated	PAM06_b	PAM06_b(B'2m)_L; PAM06_b(B'2m)_R	72	37	46	16
β′2-associated	PAM02	PAM02(B'2a)_L; PAM02(B'2a)_R	128	31	98	55
β′2-associated	PAM05	PAM05(B'2p)_L; PAM05(B'2p)_R	192	117	115	41
β2/β′2a mixed	PAM03	PAM03(B2B'2a)_L; PAM03(B2B'2a)_R	45	19	26	12
β′1	PAM13	PAM13(B'1ap)_L; PAM13(B'1ap)_R	141	92	84	32
β′1	PAM14	PAM14(B'1m)_L; PAM14(B'1m)_R	103	60	53	51
α1	PAM11	PAM11(a1)_L; PAM11(a1)_R	319	12	52	273
