# Supplementary material for: Nicotinic receptor in dopamine neurons mediates learned odor choice
Source: PLoS Genet. 2026 Oct 29;22(10):e1012356. doi: 10.1371/journal.pgen.1012356 (PMC13739223; doi:10.1371/journal.pgen.1012356)
Supplement: Supplementary file 5 [file pgen.1012356.s007.docx]

**Editors' comments:**

*The reviewers found the premise of the study to be of significant interest to the field of neuroscience and associative learning. However, they raised substantial concerns regarding the anatomical evidence, the validation of genetic tools, the presentation of physiological data, and the strength of the evidence supporting the behavioral mechanistic claims.*

*Based on the reviewer evaluations, I am inviting you to submit a substantially revised version of your manuscript that addresses reviewers' concerns. To be considered for publication, the revision must address the following requirements:*

We thank the editor and reviewers for their careful and constructive evaluation of our manuscript. We are grateful that the premise of the study was considered of significant interest, and we appreciate the clear guidance regarding the key issues that needed to be addressed. In response, we have substantially revised the manuscript by strengthening the anatomical evidence for Dα1 expression in PAM dopamine neurons, adding direct qRT-PCR validation of the Dα1 RNAi reagent and independent genetic validation of the behavioral phenotype, improving the visualization and statistical analysis of the physiological data, and tempering the mechanistic claims regarding learned odor choice. Below, we first address the four major editorial requirements and then provide detailed responses to the reviewers’ comments.

1. *Anatomical Specificity and Image Resolution: All reviewers expressed significant reservations regarding the imaging data in Figure 2. Suppressing expression in Kenyon cells using MB247-GAL80 is insufficient to rule out Dα1 expression in other MB-innervating neurons (e.g., KCs not covered by the driver, MBONs, DPM, APL). Please provide more rigorous anatomical evidence to isolate PAM expression, such as utilizing intersectional genetic approaches (e.g. split-GAL4 or FRT-STOP-FRT) or super-resolution microscopy. Meanwhile, please verify cholinergic synaptic inputs onto specific PAM dendrites and terminals, for example, by cross-referencing your anatomical claims with the adult *Drosophila* hemibrain connectome.*

We thank the editor for clearly identifying the anatomical issues that needed to be addressed. We have substantially revised this part of the manuscript.

First, we strengthened the evidence for Dα1 expression in PAM dopamine neurons and no longer rely on MB247-GAL80-mediated suppression of Kenyon-cell labeling as the primary basis for assigning Dα1 expression to PAM neurons. In the revised Fig. 2, we added an intersectional labeling experiment in which GFP expression depends on both R58E02/PAM enhancer activity and Dα1 transcriptional reporter activity. This experiment revealed labeled somata in the PAM region and neurites in MB compartments associated with reward-related PAM projections. We also revised the Results text and Fig. 2 legend to explain the cell-type-restricted endogenous Dα1-smOLLAS labeling strategy more clearly. Specifically, PAM-directed KDR.PEST removes the KDRT-flanked STOP cassette from the Dα1KDRT-STOP-smOLLAS allele, enabling anti-smOLLAS detection of endogenous Dα1-smOLLAS in KDR-expressing PAM neurons. These revisions provide a more selective anatomical basis for Dα1 expression in PAM dopamine neurons and avoid assigning Dα1 expression from MB compartment labeling alone **(Lines 122–148 and 325–331; Fig. 2 and its legend, Lines 826–848)**.

Second, we cross-referenced the anatomical interpretation with the adult Drosophila hemibrain connectome. We added a new connectome analysis summarizing predicted and curated cholinergic inputs to PAM neuron classes corresponding to the γ5, β′2, β′1, and α1 regions analyzed in this study. This analysis shows KC-to-PAM synapses within MB lobe regions and predicted or curated cholinergic inputs to PAM-side postsynaptic sites outside the MB lobes, including CRE and SMP. The results are now provided as Table S2, and the corresponding Methods section describes the hemibrain dataset, neuPrint/neuprint-python analysis, postsynaptic ROI-based classification, and neurotransmitter assignment criteria **(Lines 148–154 and 464–490; Table S2 and its caption, Lines 912–924)**.

Together, these revisions clarify the anatomical interpretation. The revised reporter and cell-type-restricted protein-labeling experiments support Dα1 expression in PAM dopamine neurons, whereas the connectome analysis provides anatomical support for cholinergic input routes to the corresponding PAM neuron classes **(Line 34; Lines 154–156 and 251–264)**.

2. *Validation of RNAi Knockdown: There is currently no validation of the Dα1 knockdown efficacy. Please provide biochemical, genetic, or physiological evidence confirming the extent of Dα1 knockdown.*

We agree that direct validation of the Dα1 RNAi reagent was required. We therefore induced the same UAS-Dα1-RNAi transgene pan-neuronally in adult flies for 3 days using tubP-GAL80ts; R57C10-GAL4 and measured Dα1 transcript levels in adult heads by qRT-PCR. Dα1 transcript levels were significantly reduced to approximately 65% of control levels (new Fig. S1) **(Lines 111–113 and 378–400; Fig. S1 and its legend, Lines 880–886; Table 1)**. These results directly validate the efficacy of the RNAi reagent, although they do not quantify knockdown specifically within PAM neurons. As independent genetic validation, PAM-directed CRISPR/Cas9 disruption of Dα1 reproduced the appetitive-memory impairment observed with Dα1 RNAi (Fig. 1C), further supporting the specificity of the Dα1-dependent behavioral phenotype **(Lines 113–116; Fig. 1C and its legend, Lines 821–823; Table 1)**.

3. *Physiological Data Visualization and Analysis: Reviewers 1 and 3 noted that the presentation of the physiological data in Figures 3 and S1 obscures the differences between genotypes. Please follow their suggestions to improve the presentation.*

In the revised manuscript, the physiological dataset previously shown in the supplementary figure has been reorganized and promoted to the main Fig. 3. The revised figure now shows genotype-separated time courses for control and Dα1-KD flies, with PAM-γ5, PAM-β′1, and PAM-α1 terminal responses displayed separately for 3-OCT, 4MCH, and sucrose stimulation. We also added clear stimulation bars, scale bars, and compartment labels to improve visual interpretation **(Lines 159–168; Fig. 3A, B and its legend, Lines 849–853)**.

We further revised the quantification panels. Instead of overlaying responses in a format that made genotype differences difficult to evaluate, the revised Fig. 3C–E now shows values from the same biological replicates and summary distributions across the pre-stimulus, stimulus, and post-stimulus epochs, displayed separately by genotype. This presentation makes clear that Dα1 knockdown attenuates odor-evoked responses in responsive PAM terminal compartments, whereas sugar-evoked responses are largely preserved under our recording conditions **(Lines 169–178; Fig. 3C–E and its legend, Lines 854–860)**.

We also revised the Fig. 3 legend, Methods, and Table 1 to clarify the statistical analysis. Specifically, epoch-based calcium-imaging data were analyzed using two-way repeated-measures ANOVA, with genotype as the between-subject factor and epoch as the within-subject factor. Significant genotype × epoch interactions were detected for 3-OCT- and 4MCH-evoked responses in both β′1 and α1 terminals. Sidak-adjusted genotype comparisons during the stimulus epoch were significant for both odors in β′1 terminals and for 3-OCT, but not 4MCH, in α1 terminals. No significant genotype × epoch interaction was detected for sugar-evoked responses. The revised Table 1 now reports the genotype × epoch interaction, epoch effect, genotype effect, and corresponding post hoc comparisons for each compartment and stimulus condition **(Lines 500–502, 509–511, and 517; Fig. 3 legend, Lines 849–860; Table 1)**.

4. *Calibration of Mechanistic Claims: Reviewer 2 raised a critical point regarding the claim that Dα1 gates "odor-driven input to support to support evaluating and selecting between competing odor options." The physiological data demonstrates that PAMs respond similarly to both OCT and MCH, and no physiological mechanism is shown for how competing signals are scaled or gated. Furthermore, the behavioral dissociation may reflect the difference in task difficulty between a single-odor approach and a two-odor forced choice, rather than a specific deficit in relative value comparison. Please provide physiological data demonstrating how PAM activity shifts during the simultaneous presentation of competing odors, or substantially revise the text to temper the mechanistic claims. Ensure that alternative interpretations, such as differences in task complexity, are thoroughly discussed.*

We thank the editor for highlighting this important concern. We agree that the original wording was too mechanistic. Our experiments show that Dα1 is required for odor-evoked PAM terminal activity and for normal learned odor-choice performance under conditions with competing odor cues, but they do not directly demonstrate how competing odor signals are scaled or gated within PAM neurons.

We therefore chose the second option suggested by the editor and substantially revised the text to temper the mechanistic claims. Throughout the Abstract, Results, and Discussion, we removed or softened wording implying that Dα1 directly “gates” odor-driven input or mediates evaluation and selection between competing odor options as a demonstrated mechanism. Instead, we now state more conservatively that Dα1-dependent nicotinic signaling supports odor-evoked PAM terminal activity and learned odor-choice performance when competing odor cues are present **(Lines 35–41, 48–50, 93–98, 181–183, 207–209, 212–229, and 251–306)**.

We also revised the behavioral interpretation to address the possibility that the observed dissociation reflects differences in task complexity between single-odor pursuit and two-odor choice. In the revised Fig. 4 and Fig. S2, we added and reorganized behavioral controls to make this point clearer, including 4MCH and 3-OCT single-odor no-choice assays, the breakdown of the performances to CS+ and CS− separately, lower-concentration no-choice assays, and innate odor-choice controls. We also report effect sizes and confidence intervals for the main no-choice comparisons in Table S1. Because the lack of a statistically significant difference does not demonstrate that no difference exists between genotypes, we no longer describe the no-choice results as “indistinguishable.” Instead, we state that Dα1 knockdown did not significantly reduce single-odor pursuit under the tested conditions **(Lines 184–209, 338–375, and 512–517; Fig. 4 and its legend, Lines 861–877; Fig. S2 and its legend, Lines 887–904; Table S1 and its caption, Lines 906–910; Table 1)**.

Finally, we revised the Discussion to make this calibration explicit while preserving the logic of the circuit discussion. In the opening Discussion summary, we now state that Dα1-dependent signaling appears particularly important under competing-odor choice conditions, but that differences in task demands and smaller effects on single-odor pursuit cannot be excluded. We also revised the end of the Discussion to identify the key experiments needed to define the downstream mechanism. Specifically, we now note that future experiments measuring dopamine release, conditioning-dependent changes in odor-evoked PAM activity, PAM responses during simultaneous presentation of competing odor cues, and odor–sugar integration will be needed to determine how Dα1-dependent PAM activity is transformed into dopamine output and choice-related behavior **(Lines 212–229 and 251–306)**.

**Reviewers' comments:**

***Reviewer #1****: The manuscript PGENETICS-D-26-00251 by Yamagata et al. titled “Nicotinic receptor in dopamine neurons mediates learned odor choice” tackles the role of nicotinic modulation of dopaminergic neurons in the context of learned odor discrimination in Drosophila. Overall, they conclude that α1 nicotinic receptor expression in reward dopaminergic neurons innervating the mushroom body is crucial to discriminate learned odor options during decision-making. I find their results interesting, compelling and clear.

I have some minor comments/suggestions of experiments that could help clarifying the data and reading/visualizing the results and discussion.*

We thank Reviewer #1 for the positive assessment of our study and for the constructive suggestions to clarify the data presentation and interpretation. We are grateful that the reviewer found the results interesting, compelling, and clear. In response to these comments, we revised the behavioral analyses and figure schematics, added missing genetic and sensory-control data, improved the presentation of the physiological data, corrected citation and reference issues, and expanded the relevant Discussion sections. We respond to each point in detail below.

*1. Although the behavioral data presented in Figure 4 are clear (though complicated to explain mechanistically), the authors should at least mention that PI1 and PI2 are not different in the way that a1 nicotinic receptor expression knock down in reward dopaminergic neurons are not affecting one of the odors (3-Oct or 4-Mch). Indeed, sparse coding is not similar between odors and within Kenyon cell subtypes. Odor discrimination in the knock down could affect one odor more than another odor. In line 150 the authors mentioned two references which does not match with the sentence. For ex, ref 79 does not have any appetitive data. Related, line 220 should add the ref 86 for MBON-b2g´1 to PAM-b´2a synapses. Figure 4 should change the water drop reward with a sugar reward schematic (or just writing “sugar” in a small box). Figure 4B shows 4-Mch avoidance, how about avoidance of 3-Oct, is that similar?*

We thank the reviewer for these helpful suggestions. We addressed each point as follows.

First, to test whether the behavioral phenotype in the two-odor choice assay was driven by one specific odor assignment, we now show the reciprocal PI1 and PI2 values separately in Supplementary Fig. S2A and S2B. In both reciprocal conditions, Dα1 knockdown in PAM dopamine neurons significantly reduced performance compared with the corresponding controls. These results indicate that the impaired learned odor choice is not attributable to a selective defect for only one of the two odors, 3-OCT or 4MCH, but is observed across both reciprocal odor assignments **(Lines 186–188 and 365–372; Fig. S2A, B and its legend, Lines 887–891; Table 1)**.

Second, in response to the reviewer’s question about the 3-OCT single-odor test, we added the corresponding 3-OCT data to complement the 4MCH data shown previously. Dα1 knockdown did not significantly impair performance in this assay, consistent with the 4MCH result. These data are now included in revised Fig. 4C **(Lines 188–196 and 346–352; Fig. 4C and its legend, Lines 864–869; Table S1 and its caption, Lines 906–910; Table 1)**.

Third, we revised the relevant Results sentence because the previous references did not directly support the statement about appetitive memory retrieval. The revised sentence now more conservatively states that Dα1-mediated odor-evoked activity in PAM dopamine neurons suggested a possible contribution to odor-guided behavioral performance rather than sugar processing per se. We also added the requested reference for the MBON-γ2α′1 to PAM-β′2a synaptic connection at the relevant sentence in the Discussion **(Lines 181–183 and 287–290)**.

Fourth, we corrected the Figure 4 schematic throughout the figure. The reward is now indicated as sugar, rather than being represented by a water-drop schematic.

*2. I am not entirely convinced by the interpretation of Figure 2. Observing a1 nicotinic receptor expression in mushroom body compartments does not mean that reward PAM neurons express them. Indeed, on the top of TH/ddc signal, Trh, tdc2 neurons and even DPM interneurons are innervating compartments of the mushroom body. The authors should dampen their conclusion (like “exclusively” in line 121 and the lines 123-124). Also check related line 195. The authors also mention g3 (line 117) but do not show it in Figure 2A. A summary schematic about the anatomical expression of PAM and a1 nicotinic receptor would be helpful (like a G panel). Related, line 80 refers to reference 58 but the conclusion is different from that of line 185-186.*

We agree that Dα1 reporter labeling in MB compartments alone is not sufficient to conclude that reward-related PAM dopamine neurons express Dα1, because MB compartments contain neurites from multiple neuronal classes. **(Lines 127–128)**

To address this concern, we added an intersectional labeling experiment in the revised Fig. 2D and 2E. In this experiment, GFP expression depends on the intersection between R58E02/PAM enhancer activity and Dα1 transcriptional reporter activity. This approach revealed labeled somata in the PAM region and neurites in MB compartments innervated by reward-related PAM neurons. We now use this intersectional experiment, rather than MB compartment labeling alone, as the main evidence that Dα1 transcriptional reporter activity is present in PAM-associated neurons. **(Lines 129–136 and 327–329; Fig. 2D, E and its legend, Lines 832–835)**

We also revised the interpretation of Fig. 2 to avoid overstatement. In particular, we softened wording that could imply exclusive PAM specificity based only on MB compartment labeling, and we revised the corresponding Discussion text accordingly. **(Lines 127–128, 141–156, and 251–264)** We also removed the reference to γ3 from the Results text because this compartment is not shown in the revised figure. In addition, we added a summary schematic in Fig. 2F to clarify the anatomical interpretation of the intersectional labeling. **(Lines 134–135; Fig. 2F and its legend, Lines 835–836)**

Finally, we revised the statements related to projection-neuron input and reference 58 to make the Introduction and Discussion consistent. The revised text now states more conservatively that direct projection-neuron input to PAM neurons is limited, rather than using this point to imply complete absence of non-MB olfactory input. **(Lines 78–85 and 251–256)**

*3. Figure 1B only shows all control groups for Da1 however, in line 106, the authors mentioned that the experiments with all control groups for Da5 could not be confirmed. The authors have to show the data. In Figure 1D, the odor avoidance is only done with one odor, which one and why not doing the other odor (see comment 1). A schematic representing the protocol used for each panel will be helpful (like done in Figure 4).*

We have added the full genetic control data for Dα5 to revised Fig. 1B. We also revised the corresponding Results text to clarify the outcome of this control experiment. The revised text now states that Dα1 and Dα5 knockdown reduced immediate memory in the initial RNAi screen, but that full genetic controls confirmed the Dα1 phenotype whereas the apparent Dα5 effect was not supported when the corresponding controls were included. **(Lines 107–111; Fig. 1A, B and its legend, Lines 814–820; Table 1)**

We also expanded the odor-avoidance controls. The original version showed the avoidance response for only one odor, but the revised manuscript now shows avoidance responses to both 3-OCT and 4MCH in revised Fig. 1D and 1E. These data indicate that Dα1 knockdown does not cause an obvious defect in innate odor avoidance to either odor used in the conditioning experiments. **(Lines 117–119 and 373–376; Fig. 1D, E and its legend, Lines 823–825; Table 1)**

Finally, we added schematic diagrams of the behavioral protocols to the relevant panels in Fig. 1, as suggested. These schematics clarify the assay design for the memory test and the sensory-control experiments. **(Fig. 1A–F and its legend, Lines 814–825)**

*4. Related to Figure 3, line 137 the authors wrote that g5 terminals weakly respond to odors while we do not see any response in Figure S1. In the same line, the authors wrote (line 138) that sugar stimulation does not induce response in β´1 while it actually leads to a small (although not significant) inhibition. Figure 3 and Figure S1 must a have a time scale, and schematic of the area imaged will be helpful.*

We have revised the Results text to better match the physiological data. Specifically, we no longer state that γ5 terminals show an odor response. Instead, we now state that odor stimulation produced clear responses in β′1 and α1 terminals, whereas γ5 terminals showed weak or no reliably detectable odor-evoked response under our recording conditions. We also revised the description of the β′1 sugar response to state that β′1 terminals did not show a significant excitatory sugar response and showed at most a small non-significant decrease. **(Lines 163–168; Fig. 3A–E and its legend, Lines 849–860; Table 1)**

We also revised the physiological-data presentation in Fig. 3. The revised figure now includes a schematic of the imaged PAM terminal regions, time/stimulation bars, and scale bars. **(Fig. 3A, B and its legend, Lines 849–854)** In addition, the quantification is now shown across pre-stimulus, stimulus, and post-stimulus epochs, allowing the genotype-dependent changes to be evaluated more clearly. **(Lines 169–178; Fig. 3C–E and its legend, Lines 854–860; Table 1)**

To support this revised analysis, we added the corresponding description to the Methods. Imaging responses are now summarized as mean log10(GCaMP6s/mCherry) values during pre-stimulus, stimulus, and post-stimulus epochs. **(Lines 500–502)** We also clarified the statistical analysis: for Fig. 3C–E, genotype effects across epochs were analyzed using two-way repeated-measures ANOVA, with genotype as the between-subject factor and epoch as the within-subject factor. **(Lines 509–511 and 517; Fig. 3C–E and its legend, Lines 854–860; Table 1)**

*5. The authors could discuss a bit more about the role of nicotinic receptors and dopamine modulation, notably citing Dr. Philippe Faure’s work (ESPCI, Paris) or this work* [*https://pmc.ncbi.nlm.nih.gov/articles/PMC6758294/*](https://pmc.ncbi.nlm.nih.gov/articles/PMC6758294/) *about the plasticity induced by nicotinic receptors.*

We thank the reviewer for this suggestion. We have added a short discussion of nicotinic regulation of dopamine signaling and dopamine-dependent plasticity, including work from Faure and colleagues and the study suggested by the reviewer. **(Lines 230–233; References 74 and 75, Lines 739–743)** We now clarify that our findings extend this broader ACh–DA modulation framework by showing that Dα1-dependent nicotinic signaling in PAM dopamine neurons preferentially supports odor-evoked synaptic activity while leaving sugar-evoked responses largely preserved. **(Lines 233–236)**

***Reviewer #2****: In this manuscript Yamagata et al. examined the role of nAChRs in PAM DANs in odor–sugar associative learning and memory. They found that the nicotinic acetylcholine receptor α1 subunit (Dα1) was detected in PAM DANs where it specifically mediated odor-evoked synaptic Ca²⁺ signals, but not sugar-evoked Ca²⁺ responses. Dα1 knockdown in PAM neurons impaired appetitive memory by reducing performance in a two-odor forced-choice discrimination task, while single-odor pursuit and innate odor choice remained intact.

The observation that nicotinic receptors are expressed in PAM DANs and contribute to synaptic responses is generally consistent with prior expectations and therefore may be of limited novelty on its own. There is one interesting result in the manuscript which is that sugar-evoked Ca²⁺ responses are not affected by Dα1 knockdown. This raises two options. Either sugar-evoked Ca²⁺ responses are not mediated by nicotinic receptors, or they are mediated by a different type of nicotinic receptors. However, the implications of this are not pursued.

Furthermore, the authors suggest that nicotinic Dα1 signaling in PAM DANs, selectively gates odor-driven input to support evaluation and selection between competing odor options. However, they provide no evidence for such gating or the underlying mechanism. According to the results presented PAMs respond the same to OCT and MCH. How then do they support evaluation and selection between competing odor options? There are a few options, but the authors do not check these.

Overall, the manuscript would benefit from a more comprehensive experimental and analytical approach. Important controls and follow-up experiments are missing, and additional analysis could substantially strengthen the conclusions. In its current form, the study does not provide sufficient depth to support its broader claims. I apologize to the authors, but I don’t think the manuscript is suitable for publication.*

We thank Reviewer #2 for the careful and critical evaluation of our manuscript. We understand the reviewer’s concern that the original manuscript did not provide sufficient anatomical specificity, validation of the Dα1 manipulation, physiological-data presentation, or mechanistic support for the broader behavioral interpretation. We have taken these concerns seriously and substantially revised the manuscript.

In the revised version, we strengthened the anatomical evidence by adding Dα1 ∩ R58E02 intersectional labeling, clarifying the cell-type-restricted endogenous Dα1-smOLLAS strategy, and adding a connectome-based summary of predicted and curated cholinergic inputs to the relevant PAM neuron classes. We also added independent genetic support for the Dα1 behavioral phenotype using PAM-directed Dα1 disruption, as well as direct qRT-PCR validation showing that the Dα1 RNAi reagent significantly reduced Dα1 transcript levels. In addition, we reorganized the physiological data so that the compartment-wide odor and sugar responses are now presented in the main Fig. 3 with genotype-separated traces and epoch-based quantification. Finally, we substantially tempered the mechanistic interpretation throughout the manuscript. We no longer claim to have demonstrated a gating mechanism for evaluating and selecting between competing odor options; instead, we state more conservatively that Dα1-dependent nicotinic signaling supports odor-evoked PAM terminal activity and learned odor-choice performance when competing odor cues are present.

We respond to each point in detail below.

*Major comments:*

*1. I am not sure I agree with the authors regarding the analysis of Dα1 expression pattern. The authors use nAChRα1-2A-GAL4 to label neurons that express Dα1. As nicotinic receptors are widely expressed in the Drosophila brain, the authors are correctly concerned from labeling neurites arising from other MB neurons. Therefore, they attempt to block expression in KCs using MB247-GAL80. However, this manipulation is not sufficient. First, GAL80 does not completely block expression (and no evidence was provided for the successful block of Dα1 expression in KCs). Second, and more importantly, the MB contains neurites of many other neurons. MBONs send dendrites to all MB compartments, are innervated by KCs, and express nicotinic receptors. APL, DPM, octopaminergic neurons all innervate MB lobes. It is true that for all these neurons cell bodies are not located in the vicinity of PAM DANs cell bodies. However, according to the authors themselves they were concerned from “obscure labeled neurites”.

A more selective strategy (e.g., intersectional approaches such as FRT-STOP-FRT) could help ensure PAM DAN-specific labeling.

The conclusion that “These results indicate that memory-inducing PAM cell classes (43, 49, 74) transcriptionally express Dα1” is based only on proximity of the labelled cell bodies to known location of PAM DANs “We detected GAL4-positive somata in the region where PAM DANs are typically located”. I don’t think this is enough.

These concerns are also relevant for the colocalization experiments. The authors use a regular confocal. However, with the dense innervation in the MB and the fact that all neurons in the circuit express nicotinic receptors, this can easily give a false positive result. For this experiment super resolution is required. This is true also for the MiMIC experiments.*

*The experiments with smOLLAS-tagged Dα1 are not explained anywhere, and it is impossible for the reader to understand that this is cell specific labelling.*

We thank the reviewer for raising this important concern. We agree that Dα1 reporter labeling in MB compartments, even with MB247-GAL80, is not sufficient by itself to assign Dα1 expression specifically to PAM dopamine neurons, because MB compartments contain neurites from multiple neuronal classes. We have therefore revised both the experimental presentation and the interpretation of Fig. 2. **(Lines 124–156; Fig. 2 and its legend, Lines 826–848)**

Most importantly, we added a new intersectional labeling experiment to more selectively identify neurons with both Dα1 transcriptional reporter activity and R58E02/PAM enhancer activity. In the revised Fig. 2, GFP expression depends on the intersection between Dα1-2A-GAL4-driven UAS-LexA.DBD and R58E02-p65.AD. This experiment revealed labeled somata in the PAM region and neurites in MB compartments associated with reward-related PAM projections. We now use this intersectional experiment as the main evidence that Dα1 transcriptional reporter activity is present in PAM dopamine neurons, rather than relying on MB compartment labeling alone. **(Lines 129–136 and 327–329; Fig. 2D–F and its legend, Lines 832–836)**

We also clarified the smOLLAS-tagged Dα1 experiment. The revised Results text and Figure 2 legend now explain that PAM-directed KDR.PEST removes the KDRT-flanked STOP cassette from the Dα1KDRT-STOP-smOLLAS allele, enabling cell-type-restricted detection of endogenous Dα1-smOLLAS in KDR-expressing PAM neurons by anti-smOLLAS immunostaining. This makes clear that the smOLLAS experiment provides cell-type-restricted protein-level evidence, distinct from the transcriptional reporter experiments. **(Lines 141–148, 154–156, and 329–331; Fig. 2I–L and its legend, Lines 839–848)**

Accordingly, we have softened the anatomical interpretation throughout the manuscript. We no longer infer PAM-specific Dα1 expression from MB247-GAL80 labeling or from dense MB neuropil colocalization alone, and we avoid implying that every Dα1 punctum in the MB neuropil can be assigned to PAM neurons. Instead, the revised conclusion is based on complementary evidence from intersectional reporter labeling and cell-type-restricted endogenous Dα1 protein visualization. **(Lines 127–156; Fig. 2D–L and its legend, Lines 832–848)**

*The figure quality also makes evaluation challenging:
a. Figure 2A: Where are PAM DANs somata located? PAM DANs should be labeled with anti-TH, or at least their presumed locations should be visually marked.*

We have revised Fig. 2A and 2B to explicitly show where the PAM soma regions are located. The revised figure now includes a broader protocerebral view and a magnified optical section from nAChRα1-2A-GAL4, MB247-GAL80/UAS-mCD8::GFP flies. The anatomical PAM soma regions are visually marked in the magnified panel, as requested, and the Figure 2 legend has been revised accordingly. **(Lines 124–126; Fig. 2A, B and its legend, Lines 826–830)**

*b. Figures 2B-2C: The picture is not clear - the MB structure, and specifically the gamma lobe, cannot be identified. In addition, the region in Figure 2B, magnified in Figure 2C, should be visually demarcated and explicitly described in the Figure legend.*

We have revised the corresponding panels, now shown as Fig. 2G and 2H, to make the MB γ-lobe region clearer. In the revised Fig. 2G, the γ5 region within the γ lobe is indicated by a white dashed outline, and the region magnified in Fig. 2H is demarcated by a box. We also revised the Figure 2 legend to explicitly state that Fig. 2H shows a magnified view of the boxed region in Fig. 2G. **(Lines 137–143; Fig. 2G, H and its legend, Lines 836–839)**

*c. Figure 2D-2F: The picture is not clear. The magnified figures do not seem to be magnification of the low magnification image. In addition, it is unclear how the authors identified axonal terminals/dendrites. Lastly, the detected signals in Figures 2D-2E are not observed in the MB, in contrast to Figures 2A-2C.*

We have revised the corresponding panels, now shown as Fig. 2I–L, and clarified their description in the Figure 2 legend. The revised Fig. 2I now explains the genetic logic of the cell-type-restricted Dα1-smOLLAS labeling strategy. Specifically, PAM-directed KDR.PEST removes the KDRT-flanked STOP cassette from the Dα1KDRT-STOP-smOLLAS allele, allowing endogenous Dα1-smOLLAS to be detected selectively in KDR-expressing PAM neurons by anti-smOLLAS immunostaining. **(Lines 143–148 and 329–331; Fig. 2I and its legend, Lines 839–842)**

We also clarified the anatomical regions shown in the corresponding image panels. Fig. 2J and 2K show MB-extrinsic PAM neurites in the crepine and superior medial protocerebrum, corresponding to putative dendritic/input-enriched regions, whereas Fig. 2L shows PAM axon-terminal regions within the MB lobes. We therefore no longer present these panels as simple magnifications of a single low-magnification image, but describe them as representative optical sections from the indicated regions. **(Lines 147–153; Fig. 2J–L and its legend, Lines 842–848)**

*2. The manuscript is missing important controls. For example, there is no validation of Dα1 knockdown, not even at the level of the whole brain. There is no attempt to examine acute ACh induced responses. In addition, Dα1 knockdown is performed throughout development. There is no examination that the results do not arise from developmental effects.*

We thank the reviewer for raising these important concerns regarding RNAi validation, acute acetylcholine responsiveness, and possible developmental effects.

First, we have now directly validated the efficacy of the UAS-Dα1-RNAi reagent. The same RNAi transgene used in our behavioral and physiological experiments was induced pan-neuronally for 3 days specifically in adult flies using tubP-GAL80ts; R57C10-GAL4, and Dα1 transcript levels were measured by qRT-PCR using adult heads. Dα1 transcript levels were significantly reduced to approximately 65% of control levels (new Fig. S1). We present this experiment as validation of RNAi efficacy rather than as a quantitative measurement of knockdown specifically within PAM neurons. **(Lines 111–113 and 378–400; Fig. S1 and its legend, Lines 880–886; Table 1)**

Second, we tested the behavioral requirement for Dα1 using an independent genetic strategy. PAM-directed CRISPR/Cas9 disruption of Dα1, using R58E02-GAL4-driven Cas9 together with a Dα1 sgRNA, reproduced the appetitive-memory impairment observed with Dα1 RNAi (Fig. 1C). This independent genetic replication makes it unlikely that the behavioral phenotype results from an off-target effect specific to the RNAi reagent. **(Lines 113–116; Fig. 1C and its legend, Lines 821–823; Table 1)**

Regarding acute ACh-induced responses, we agree that we did not directly apply exogenous ACh in the present study. However, acute ACh responsiveness of dopaminergic terminals in the adult mushroom body has previously been demonstrated directly using fast-scan cyclic voltammetry. Acute ACh application to the PAM-innervated medial tip of the adult mushroom body evoked rapid dopamine release that returned toward baseline within approximately 10 s (Shin and Venton, 2022; ref. 58; see also ref. 57). We have made this prior evidence more explicit in the revised manuscript. **(Lines 83–85; References 57 and 58, Lines 687–692)** Rather than repeating the general demonstration that acute ACh can evoke dopamine release, the present experiments examined whether Dα1 contributes to PAM-terminal responses evoked by behaviorally relevant odor and sugar stimulation. **(Lines 159–178; Fig. 3 and its legend, Lines 849–860; Table 1)** We nevertheless acknowledge that our experiments do not directly determine the contribution of Dα1 to dopamine release evoked by exogenous ACh.

Finally, we agree that the constitutive PAM-directed RNAi and CRISPR/Cas9 experiments do not formally exclude developmental contributions, and we now state this limitation in the Discussion. **(Lines 237–241)** The adult-specific qRT-PCR experiment demonstrates that the same RNAi reagent can reduce Dα1 expression after development, although it does not establish that adult-specific knockdown is sufficient to produce the behavioral phenotype. **(Lines 111–113 and 378–400; Fig. S1 and its legend, Lines 880–886; Table 1)** Nevertheless, the selective attenuation of odor-evoked responses in adult PAM terminals, together with preserved sugar-evoked responses and normal odor avoidance and sugar attraction, demonstrates a specific functional consequence in the mature circuit and argues against a generalized developmental or sensory deficit. **(Lines 117–119, 169–178, and 237–241; Fig. 1D–F and its legend, Lines 823–825; Fig. 3C–E and its legend, Lines 854–860; Table 1)** Thus, our data support a requirement for Dα1 in normal mature PAM circuit function, while adult-restricted behavioral manipulation will be needed to determine the timing of this requirement. **(Lines 237–241)**

*3. The authors’ main claim is that Dα1 is required for odors but not sugar induced responses. This is an interesting result. However, the authors do not examine its importance. Are sugar responses mediated by a different neurotransmitter? Are they mediated by the cholinergic muscarinic receptors? Are they mediated by a different nicotinic receptor? Do sugar induced responses have different properties than odor induced responses? Different kinetics? Do they last for longer? Given the relatively limited scope of the results presented, a more thorough examination of these questions would be expected.

The presented data could be improved. The authors claim that Dα1 knockdown reduces odor induced responses but not sugar induced responses. They choose to show the results obtained from alpha1. However, in this brain region only 3-OCT has a significant effect, whereas MCH was not significant, like the sugar.*

*I guess the authors wanted to show in the same brain region the differential effect. However, this claim is better supported in Figure S1, where across brain regions Sugar response is not affected whereas odor responses are generally affected.

I think it is best to discard current Figure 3 and make Figure S1 new Figure 3. In particular as Figure 3A is identical to the data presented in Figures S1A and S1B. In addition, Figures 3B and 3C are identical to the data presented in Figure S1C.

Scale bar should be located near the traces in Figure S1A and S1B and described in the Figure legend.*

We thank the reviewer for these helpful comments. We agree that the mechanism underlying the Dα1-independent sugar response is an important open question. **(Lines 242–250)** The present study was designed to test whether Dα1 is required for odor- and sugar-evoked PAM terminal responses, rather than to define the full receptor or transmitter mechanism underlying the sugar pathway. We have therefore revised the text to state our conclusion more precisely: Dα1 knockdown attenuates odor-evoked PAM terminal responses, whereas sugar-evoked responses are preserved under our recording conditions. **(Lines 93–94, Lines 163–178 and 212–229; Fig. 3 and its legend, Lines 849–860; Table 1)** We do not claim to identify the receptor or transmitter mechanism that mediates the remaining sugar response. **(Lines 242–250)**

We also considered the reviewer’s suggestion to compare the properties of odor- and sugar-evoked responses. However, odor and sugar stimulation differ in sensory modality, delivery route, and the PAM compartments that respond most strongly. We therefore focused the revised analysis on whether each stimulus evoked detectable PAM terminal responses and whether those responses depended on Dα1, rather than making a direct kinetic comparison between odor- and sugar-evoked responses. **(Lines 159–178; Fig. 3 and its legend, Lines 849–860; Table 1)** We have expanded the Discussion to note that sugar-evoked PAM activity could be mediated by other nicotinic receptor subunits, muscarinic receptors, or non-cholinergic reward-related inputs. **(Lines 242–250)** As one example, we now cite larval work showing that Kenyon-cell-to-pPAM feedback can support appetitive reward signaling through short neuropeptide F rather than acetylcholine, illustrating that reward-related DAN modulation need not be exclusively cholinergic. **(Lines 246–250; Reference 76, Lines 744–746)**

We also revised the presentation of the physiological data as suggested. The dataset previously shown in Fig. S1 has been promoted to the revised main Fig. 3, replacing the previous α1-focused presentation with a compartment-wide analysis of PAM-γ5, PAM-β′1, and PAM-α1 responses. **(Lines 159–178; Fig. 3A–E and its legend, Lines 849–860)** Scale bars are now placed near the traces and described in the Figure 3 legend. **(Fig. 3A, B and its legend, Lines 849–854)** Quantification is shown separately by genotype across the pre-stimulus, stimulus, and post-stimulus epochs, with values from the same biological replicates analyzed by two-way repeated-measures ANOVA. **(Lines 500–511 and 517; Fig. 3C–E and its legend, Lines 854–860; Table 1)** Significant genotype × epoch interactions were detected for both 3OCT- and 4MCH-evoked responses in β′1 and α1 terminals. Sidak-adjusted comparisons within individual epochs showed significantly lower signals in Dα1-KD flies during the stimulus epoch for both odors in β′1 terminals and for 3OCT in α1 terminals; the corresponding 4MCH comparison in α1 terminals did not reach significance. By contrast, no significant genotype × epoch interaction was detected for sugar-evoked responses in any of these compartments. Complete statistical results are provided in Table 1. Thus, the revised analysis indicates that Dα1 knockdown alters odor-evoked PAM terminal response profiles, whereas sugar-evoked responses are largely preserved under our recording conditions. **(Lines 169–178; Fig. 3C–E and its legend, Lines 854–860; Table 1)**

*4. The authors’ second main claim is that Dα1 is required to selectively gate odor-driven input to support evaluation and selection between competing odor options. However, the authors do not show such gating, nor do they provide evidence on how it is achieved. Is summation of the sugar and odor response required? Is there a change in odor induced signals following conditioning? Is DA release affected?*

We agree that the original wording was too mechanistic. Our experiments show that Dα1 is required for odor-evoked PAM terminal activity and for normal learned odor-choice performance, but they do not directly demonstrate the downstream mechanism by which this signal contributes to evaluation or selection between competing odor options. **(Lines 169–178, 184–209, and 265–306; Fig. 3 and its legend, Lines 849–860; Fig. 4 and its legend, Lines 861–877; Table 1)**

We have therefore revised the Abstract, Results, and Discussion to avoid implying that we have demonstrated the gating mechanism itself. In particular, we no longer state that Dα1 “selectively gates odor-driven input to support evaluation and selection” as a demonstrated mechanism. Instead, we state more conservatively that Dα1-dependent nicotinic signaling supports odor-evoked PAM terminal activity and learned odor-choice performance when competing odor cues are present. **(Lines 35–41, 93–98, 181–183, 207–209, 212–229, and 265–306)**

We also revised the Discussion to clarify the remaining mechanistic questions while preserving the logic of the circuit discussion. The revised circuit discussion now asks how Dα1-dependent odor-evoked PAM activity could be linked to learned odor-choice performance at the circuit level, and then considers KC–PAM and MBON-guided circuit routes as possible mechanisms. **(Lines 251–298)** At the end of this discussion, we now state that Dα1-dependent odor-evoked PAM terminal activity provides a cellular entry point into learned odor choice, while the downstream mechanism remains to be defined. We further note that future experiments measuring dopamine release, conditioning-dependent changes in odor-evoked PAM activity, PAM responses during simultaneous presentation of competing odor cues, and odor–sugar integration will be needed to determine how this activity is transformed into dopamine output and choice-related behavior. **(Lines 299–306)**

*5. The no-choice result in Figure 4B is supported only by non-significant differences without equivalence testing, effect sizes, confidence intervals, or power analysis to justify an 'indistinguishable' conclusion. Furthermore, this is an easier task than the choice task, especially as high odor concentrations are used (10-fold dilution). Small but meaningful deficits could be missed. It would have been better to examine the no-choice test under more difficult conditions using lower concentrations or odor mixtures.*

We thank the reviewer for this important point. We agree that a non-significant difference alone does not establish equivalence, and we have revised the wording accordingly. We no longer describe no-choice performance as “indistinguishable.” Instead, we state that Dα1 knockdown did not significantly reduce single-odor no-choice performance under the tested conditions. **(Lines 188–190 and 220–224; Fig. 4B, C and its legend, Lines 864–869; Table 1)**

We also added no-choice tests using 3OCT as well as 4MCH. **(Lines 188–190; Fig. 4B, C and its legend, Lines 864–869; Table 1)** For these main no-choice comparisons, we now report effect sizes and confidence intervals for Dα1-KD versus each genetic control in Table S1. **(Lines 193–194 and 512–516; Table S1 and its caption, Lines 906–910)** In addition, to address the concern that the no-choice assay may be easier at high odor concentration, we performed additional no-choice tests using lower odor concentrations (Figs. S2E, S2F, S2H). Dα1 knockdown or knockout also did not significantly reduce performance under these lower-concentration conditions. **(Lines 195–196; Fig. S2E, F, H and its legend, Lines 896–904; Table 1)** We also revised the opening Discussion summary to state that Dα1-dependent signaling appears particularly important under competing-odor choice conditions, while acknowledging that differences in task demands and smaller effects on single-odor pursuit cannot be excluded. **(Lines 219–224)**

*6. Figure legends lack sufficient detail in several cases, making it difficult to fully interpret the data.*

We have revised the figure legends throughout the manuscript to provide more information needed to interpret the data. In particular, we expanded the Fig. 2 legend to explain the Dα1 ∩ R58E02 intersectional labeling, the cell-type-restricted Dα1-smOLLAS strategy, the anatomical regions shown in the smOLLAS panels, and the relationship between boxed and magnified regions. **(Fig. 2 legend, Lines 826–848)** We also revised the Fig. 3 legend to describe the genotype-separated traces, stimulus period, scale bars, epoch-based quantification, and statistical analysis. **(Fig. 3 legend, Lines 849–860)** The Fig. 4 and Fig. S2 legends were also revised to clarify the behavioral protocols, odor conditions, biological replicates, and statistical tests. **(Fig. 4 legend, Lines 861–877; Fig. S2 legend, Lines 887–904)**

*Minor comments:

1. I did not find in Figure 1B the experiments referring to Dα5.*

We thank the reviewer for pointing this out. We have added the Dα5 genetic-control data to the revised Fig. 1B. The revised panel now shows that the apparent Dα5 effect observed in the initial RNAi screen was not confirmed when the corresponding genetic controls were included. **(Lines 107–111; Fig. 1B and its legend, Lines 817–820; Table 1)**

*2. There are some errors in the reference list:
a. References 31 and 89 refer to the same manuscript.*

We have revised. **(Reference 31, Lines 616–618)**

*b. Reference 98 is wrongly formatted “Drosophila”.*

We have revised. **(Reference 98, Lines 806–808)**

*c. Reference 87 was retracted and cannot be cited unless explicitly marking it as retracted and only if not used as evidence as it currently is.*

We thank the reviewer for carefully pointing this out. We have now removed the retracted citation and revised the relevant text so that no claim relies on it. **(Lines 225–226)**

*3. I think there is a mistake in Figure 4B protocol bottom. I think the test should be OCT.*

We thank the reviewer for raising this point. The use of MCH as the test odor in both reciprocal conditions was intentional, but we agree that the rationale was not sufficiently clear in the original schematic and legend.

In this no-choice assay, the goal was to test the response to the same odor after it had been experienced either as sucrose-paired or sucrose-unpaired during reciprocal training. Thus, MCH was tested in both reciprocal groups to keep the test-odor identity constant and avoid confounds from odor-specific innate preference, intensity, volatility, or sensorimotor responses. We have revised the Figure 4B schematic, legend, and Behavioral assays section to make this design clearer. **(Lines 346–352; Fig. 4B and its legend, Lines 864–868)**

To further confirm that the no-choice result was not specific to MCH, we also added a complementary OCT-centered no-choice experiment using the same logic, now shown in Fig. 4C. **(Lines 188–190 and 346–352; Fig. 4C and its legend, Lines 866–869; Table 1)** In addition, we added new component plots in Fig. S2C and S2D, in which the same no-choice data are re-plotted according to training contingency. These panels show the responses to the sucrose-paired odor (CS+) and the unpaired odor (CS−) separately, clarifying how the no-choice learning indices in Fig. 4B and 4C were derived from reciprocal paired and unpaired training conditions. **(Lines 191–193 and 365–372; Fig. S2C, D and its legend, Lines 891–896; Table 1)**

*4. While I understand the use of log(GCamP/mCherry), this is less intuitive. Perhaps it would be useful to show also DF/F.*

We thank the reviewer for this helpful suggestion. We agree that ΔF/F is often more intuitive for visualizing calcium responses. In this study, however, we used a ratiometric active-zone calcium sensor and therefore analyzed responses consistently as log10(GCaMP6s/mCherry). **(Lines 161–163 and 493–502; Fig. 3 and its legend, Lines 849–860)** We chose not to add a separate ΔF/F representation because it would introduce an additional transformation and baseline normalization while conveying the same response information.

Instead, we revised Fig. 3 to make the log-ratio data easier to interpret. The revised figure now shows genotype-separated traces, the stimulation period, scale bars, and pre-stimulus, stimulus, and post-stimulus quantification. **(Lines 159–178; Fig. 3 and its legend, Lines 849–860)** We also clarified the statistical analysis in the figure legend and Table 1. **(Lines 509–511 and 517; Fig. 3 legend, Lines 857–860; Table 1)**

*5. The behavioral memory index (LI) is not explained in the methods section.*

We have revised the Behavioral assays section to explicitly define the preference index and learning index. The revised Methods now state how PI was calculated for each test and that LI was calculated from the two reciprocal training groups as (PI1 − PI2)/2, with positive values indicating learned preference for the sucrose-paired odor. **(Lines 365–372)**

*6. The odor generalization experiment is not explained well. The test protocol illustration is unclear. For PI1: the authors presented sugar with the odor, for PI2, they presented the sugar after the odor (and didn’t mention the ISI). It is not clear how this manipulation is related to the question. More importantly, this contrasts with the method explanation: “For the odor generalization protocol (Fig. 4E), flies were trained with an odor for 1 minute in a constant air stream, with or without dried 2M sucrose paper. The conditioned response of the trained flies was subsequently measured.”*

We thank the reviewer for pointing out that the odor-generalization protocol was not described clearly enough. The delayed sucrose presentation in the unpaired condition was intentional and follows the standard paired versus unpaired appetitive-conditioning design, in which the odor and reinforcement are temporally separated in the unpaired condition. However, we agree that the timing relationship and ISI were not sufficiently clear in the original Methods and schematic. **(Lines 342–344 and 358–361)**

We have therefore revised the Behavioral assays section to state generally that training stimulus presentations were separated by a 1-min interstimulus interval, including odor-to-odor, odor-to-sucrose, and sucrose-to-odor intervals, as applicable. **(Lines 342–344)** We also revised the odor-generalization description to clarify that flies were trained with 4MCH using paired and unpaired sucrose protocols and were then tested with mixtures of 4MCH and the similar odor 2MCH at the indicated ratios. **(Lines 357–364; Fig. 4E and its legend, Lines 871–877)**

*7. Line 111: I think it should be “supports” (not “support”).*

We have revised. **(Line 122)**

*8. Table 1:
Figure 1A: The Brown-Forsythe test is used to test for the equality of group variances. Was ANOVA conducted? If so, what was the p-value?

The text depicting the statistical test results is not aligned with the table rows. b. ** should be in the row below.*

*Figures 3B-C: Was a repeated measures 2-way ANOVA conducted?

In general, the main statistical tests used are not explicitly mentioned (e.g., ANOVA, Kruskal-Wallis, t-test, Mann-Whitney), in contrast to the post-hoc tests.

Figure S1 is not listed.*

We thank the reviewer for these careful comments. We have revised Table 1 to make the statistical analyses clearer and to correct the formatting errors. **(Table 1)**

For Fig. 1A, we now explicitly report both the Brown-Forsythe test result and the main one-way ANOVA result, followed by Dunnett’s adjusted post-hoc comparisons. We also corrected the previous misalignment of the significance notation in the post-hoc test results. **(Fig. 1A legend, Lines 814–817; Table 1)**

For Fig. 3, we clarified that the epoch-based calcium-imaging analyses were performed using two-way repeated-measures ANOVA, with genotype as the between-subject factor and epoch as the within-subject factor. The revised table now reports the genotype × epoch interaction, epoch effect, and genotype effect, together with the corresponding post-hoc comparisons. **(Lines 509–511 and 517; Fig. 3 legend, Lines 857–860; Table 1)**

More generally, we revised Table 1 so that the main statistical test used for each figure panel is explicitly listed, including one-way ANOVA, Kruskal-Wallis test, two-way ANOVA, or two-way repeated-measures ANOVA, as appropriate. **(Lines 504–517; Table 1)** We also added the statistical results for all Fig. S1 and Fig. S2 panels. **(Fig. S1 legend, Lines 880–886; Fig. S2 legend, Lines 887–904; Table 1)**

***Reviewer #3****: This manuscript details the role of nicotinic acetylcholine receptors in dopaminergic PAM neurons in mediating olfactory input during appetitive learning. The story is succinct, the data look good overall, the study includes key controls, and the conclusions/interpretations are supported by the data. The results of the study will add to our understanding of how the circuit functions during reward learning. There are two major aspects of the data presentation that need to be addressed prior to publication.*

We thank Reviewer #3 for the positive assessment of our study and for the constructive suggestions regarding data presentation. We are grateful that the reviewer found the story succinct, the data generally strong, and the conclusions supported by the results. In response to the reviewer’s two main concerns, we revised Fig. 2 to make the evidence for Dα1 expression in PAM dopamine neurons clearer and revised Fig. 3 to improve visualization of genotype-dependent physiological differences. Specifically, we added Dα1 ∩ R58E02 intersectional labeling, clarified the cell-type-restricted Dα1-smOLLAS strategy, and reorganized the physiological data into genotype-separated traces and epoch-based quantification. We respond to each point in detail below.

*In figure 2, subcellular colocalization of the Dalpha1 receptor and TH is not clear. There is clear punctate labeling of both. What is the evidence that these are in the same cells? This should be described and pointed out clearly in the figure.*

We thank the reviewer for this helpful comment. We agree that punctate Dα1 MiMiC and TH signals in the dense MB neuropil are difficult to interpret by colocalization alone. We have therefore revised Fig. 2 and the accompanying text so that the evidence for PAM-cell association does not rely solely on apparent Dα1/TH colocalization. **(Lines 137–156; Fig. 2G–L and its legend, Lines 836–848)**

Specifically, we added an intersectional labeling experiment in which GFP expression depends on the intersection between Dα1 transcriptional reporter activity and the R58E02/PAM enhancer. **(Lines 129–136 and 327–329; Fig. 2D–F and its legend, Lines 832–836)** We also clarified the cell-type-restricted Dα1-smOLLAS experiment, in which PAM-directed KDR.PEST enables selective anti-smOLLAS detection of endogenous Dα1 in KDR-expressing PAM neurons. **(Lines 143–148, 154–156, and 329–331; Fig. 2I–L and its legend, Lines 839–848)** Together, these revisions make the evidence for Dα1 expression in PAM dopamine neurons clearer in the figure and text.

*In the physiological/imaging data presented in figures 3B,C and S1, individual responses for both genotypes are intermingled, making it difficult to see the differences. The data should be presented in a more standard format (e.g., box plots for each genotype / time, and/or individual data points + bar graphs [as in the presentation of behavioral data in figure 4]).*

We have revised the physiological-data presentation in Fig. 3 to avoid intermingling responses from the two genotypes. The dataset previously shown in Fig. S1 has been promoted to the revised main Fig. 3, and the quantification panels are now shown in a standard genotype-separated format, with individual data points overlaid on box plots for each pre-stimulus, stimulus, and post-stimulus epoch. This revised format makes the genotype-dependent reduction in odor-evoked responses easier to evaluate. **(Lines 169–178 and 500–511; Fig. 3C–E and its legend, Lines 854–860; Table 1)** We also added a schematic of the imaged PAM terminal regions, time/stimulation bars, and scale bars to the revised figure. **(Fig. 3A, B and its legend, Lines 849–854)**
