# Supplementary material for: Nicotinic receptor in dopamine neurons mediates learned odor choice
Source: PLoS Genet. 2026 Oct 29;22(10):e1012356. doi: 10.1371/journal.pgen.1012356 (PMC13739223; doi:10.1371/journal.pgen.1012356)
Supplement: Peer review history

# Peer review: Decision Letter 0

**DOI:** 10.1371/journal.pgen.1012356.r001

**Authors:** Pablo Wappner, Chun Han

4 May 2026

PGENETICS-D-26-00251

Nicotinic receptor in dopamine neurons mediates learned odor choice

PLOS Genetics

Dear Dr. Yamagata,

Thank you for submitting your manuscript to PLOS Genetics. Your study presents an intriguing uncoupling of odor-evoked and sugar-evoked calcium responses in PAM dopamine neurons, suggesting a novel, modality-specific cholinergic input channel into reward-signaling circuits.

The reviewers found the premise of the study to be of significant interest to the field of neuroscience and associative learning. However, they raised substantial concerns regarding the anatomical evidence, the validation of genetic tools, the presentation of physiological data, and the strength of the evidence supporting the behavioral mechanistic claims.

Based on the reviewer evaluations, I am inviting you to submit a substantially revised version of your manuscript that addresses reviewers' concerns. To be considered for publication, the revision must address the following requirements:

1. Anatomical Specificity and Image Resolution: All reviewers expressed significant reservations regarding the imaging data in Figure 2. Suppressing expression in Kenyon cells using MB247-GAL80 is insufficient to rule out Dα1 expression in other MB-innervating neurons (e.g., KCs not covered by the driver, MBONs, DPM, APL). Please provide more rigorous anatomical evidence to isolate PAM expression, such as utilizing intersectional genetic approaches (e.g. split-GAL4 or FRT-STOP-FRT) or super-resolution microscopy. Meanwhile, please verify cholinergic synaptic inputs onto specific PAM dendrites and terminals, for example, by cross-referencing your anatomical claims with the adult *Drosophila* hemibrain connectome.

2. Validation of RNAi Knockdown: There is currently no validation of the Dα1 knockdown efficacy. Please provide biochemical, genetic, or physiological evidence confirming the extent of Dα1 knockdown.

3. Physiological Data Visualization and Analysis: Reviewers 1 and 3 noted that the presentation of the physiological data in Figures 3 and S1 obscures the differences between genotypes. Please follow their suggestions to improve the presentation.

4. Calibration of Mechanistic Claims: Reviewer 2 raised a critical point regarding the claim that Dα1 gates "odor-driven input to support to support evaluating and selecting between competing odor options." The physiological data demonstrates that PAMs respond similarly to both OCT and MCH, and no physiological mechanism is shown for how competing signals are scaled or gated. Furthermore, the behavioral dissociation may reflect the difference in task difficulty between a single-odor approach and a two-odor forced choice, rather than a specific deficit in relative value comparison. Please provide physiological data demonstrating how PAM activity shifts during the simultaneous presentation of competing odors, or substantially revise the text to temper the mechanistic claims. Ensure that alternative interpretations, such as differences in task complexity, are thoroughly discussed.

Please submit your revised manuscript by Jul 03 2026 11:59PM. If you will need more time than this to complete your revisions, please reply to this message or contact the journal office at plosgenetics@plos.org. When you're ready to submit your revision, log on to https://www.editorialmanager.com/pgenetics/ and select the 'Submissions Needing Revision' folder to locate your manuscript file.

Please include the following items when submitting your revised manuscript:

* A letter that responds to each point raised by the editor and reviewer(s). You should upload this letter as a separate file labeled 'Response to Reviewers'. This file does not need to include responses to any formatting updates and technical items listed in the 'Journal Requirements' section below.

* A marked-up copy of your manuscript that highlights changes made to the original version. You should upload this as a separate file labeled 'Revised Manuscript with Track Changes'.

* An unmarked version of your revised paper without tracked changes. You should upload this as a separate file labeled 'Manuscript'.

If you would like to make changes to your financial disclosure, competing interests statement, or data availability statement, please make these updates within the submission form at the time of resubmission. Guidelines for resubmitting your figure files are available below the reviewer comments at the end of this letter.

We look forward to receiving your revised manuscript.

Kind regards,

Chun Han, Ph.D.

Academic Editor

PLOS Genetics

Pablo Wappner

Section Editor

PLOS Genetics

Aimée Dudley

Editor-in-Chief

PLOS Genetics

Anne Goriely

Editor-in-Chief

PLOS Genetics

**Journal Requirements:**

1) We ask that a manuscript source file is provided at Revision. Please upload your manuscript file as a .doc, .docx, .rtf or .tex. If you are providing a .tex file, please upload it under the item type u2018LaTeX Source Fileu2019 and leave your .pdf version as the item type u2018Manuscriptu2019.

2) Please upload all main figures as separate Figure files in .tif or .eps format. For more information about how to convert and format your figure files please see our guidelines:

https://journals.plos.org/plosgenetics/s/figures

3) We notice that your supplementary Figures are included in the manuscript file. Please remove them and upload them with the file type 'Supporting Information'. Please ensure that each Supporting Information file has a legend listed in the manuscript after the references list.

4) Please amend your detailed Financial Disclosure statement. This is published with the article. It must therefore be completed in full sentences and contain the exact wording you wish to be published.

State what role the funders took in the study. If the funders had no role in your study, please state: "The funders had no role in study design, data collection and analysis, decision to publish, or preparation of the manuscript.".

If you did not receive any funding for this study, please simply state: u201cThe authors received no specific funding for this work.u201d

**Reviewers' comments:**

Reviewer’s Responses to Questions

**Comments to the Authors:**

**Please note here if the review is uploaded as an attachment.**

Reviewer #1: The manuscript PGENETICS-D-26-00251 by Yamagata et al. titled “Nicotinic receptor in dopamine neurons mediates learned odor choice” tackles the role of nicotinic modulation of dopaminergic neurons in the context of learned odor discrimination in Drosophila. Overall, they conclude that �1 nicotinic receptor expression in reward dopaminergic neurons innervating the mushroom body is crucial to discriminate learned odor options during decision-making. I find their results interesting, compelling and clear.

I have some minor comments/suggestions of experiments that could help clarifying the data and reading/visualizing the results and discussion.

1. Although the behavioral data presented in Figure 4 are clear (though complicated to explain mechanistically), the authors should at least mention that PI1 and PI2 are not different in the way that a1 nicotinic receptor expression knock down in reward dopaminergic neurons are not affecting one of the odors (3-Oct or 4-Mch). Indeed, sparse coding is not similar between odors and within Kenyon cell subtypes. Odor discrimination in the knock down could affect one odor more than another odor. In line 150 the authors mentioned two references which does not match with the sentence. For ex, ref 79 does not have any appetitive data. Related, line 220 should add the ref 86 for MBON-b2g´1 to PAM-b´2a synapses. Figure 4 should change the water drop reward with a sugar reward schematic (or just writing “sugar” in a small box). Figure 4B shows 4-Mch avoidance, how about avoidance of 3-Oct, is that similar?

2. I am not entirely convinced by the interpretation of Figure 2. Observing a1 nicotinic receptor expression in mushroom body compartments does not mean that reward PAM neurons express them. Indeed, on the top of TH/ddc signal, Trh, tdc2 neurons and even DPM interneurons are innervating compartments of the mushroom body. The authors should dampen their conclusion (like “exclusively” in line 121 and the lines 123-124). Also check related line 195. The authors also mention g3 (line 117) but do not show it in Figure 2A. A summary schematic about the anatomical expression of PAM and a1 nicotinic receptor would be helpful (like a G panel).

Related, line 80 refers to reference 58 but the conclusion is different from that of line 185-186.

3. Figure 1B only shows all control groups for Da1 however, in line 106, the authors mentioned that the experiments with all control groups for Da5 could not be confirmed. The authors have to show the data. In Figure 1D, the odor avoidance is only done with one odor, which one and why not doing the other odor (see comment 1). A schematic representing the protocol used for each panel will be helpful (like done in Figure 4).

4. Related to Figure 3, line 137 the authors wrote that g5 terminals weakly respond to odors while we do not see any response in Figure S1. In the same line, the authors wrote (line 138) that sugar stimulation does not induce response in �´1 while it actually leads to a small (although not significant) inhibition. Figure 3 and Figure S1 must a have a time scale, and schematic of the area imaged will be helpful.

5. The authors could discuss a bit more about the role of nicotinic receptors and dopamine modulation, notably citing Dr. Philippe Faure’s work (ESPCI, Paris) or this work https://pmc.ncbi.nlm.nih.gov/articles/PMC6758294/ about the plasticity induced by nicotinic receptors.

Reviewer #2: In this manuscript Yamagata et al. examined the role of nAChRs in PAM DANs in odor–sugar associative learning and memory. They found that the nicotinic acetylcholine receptor α1 subunit (Dα1) was detected in PAM DANs where it specifically mediated odor-evoked synaptic Ca²⁺ signals, but not sugar-evoked Ca²⁺ responses. Dα1 knockdown in PAM neurons impaired appetitive memory by reducing performance in a two-odor forced-choice discrimination task, while single-odor pursuit and innate odor choice remained intact.

The observation that nicotinic receptors are expressed in PAM DANs and contribute to synaptic responses is generally consistent with prior expectations and therefore may be of limited novelty on its own. There is one interesting result in the manuscript which is that sugar-evoked Ca²⁺ responses are not affected by Dα1 knockdown. This raises two options. Either sugar-evoked Ca²⁺ responses are not mediated by nicotinic receptors, or they are mediated by a different type of nicotinic receptors. However, the implications of this are not pursued.

Furthermore, the authors suggest that nicotinic Dα1 signaling in PAM DANs, selectively gates odor-driven input to support evaluation and selection between competing odor options. However, they provide no evidence for such gating or the underlying mechanism. According to the results presented PAMs respond the same to OCT and MCH. How then do they support evaluation and selection between competing odor options? There are a few options, but the authors do not check these.

Overall, the manuscript would benefit from a more comprehensive experimental and analytical approach. Important controls and follow-up experiments are missing, and additional analysis could substantially strengthen the conclusions. In its current form, the study does not provide sufficient depth to support its broader claims. I apologize to the authors, but I don’t think the manuscript is suitable for publication.

Major comments:

1. I am not sure I agree with the authors regarding the analysis of Dα1 expression pattern. The authors use nAChRα1-2A-GAL4 to label neurons that express Dα1. As nicotinic receptors are widely expressed in the Drosophila brain, the authors are correctly concerned from labeling neurites arising from other MB neurons. Therefore, they attempt to block expression in KCs using MB247-GAL80. However, this manipulation is not sufficient. First, GAL80 does not completely block expression (and no evidence was provided for the successful block of Dα1 expression in KCs). Second, and more importantly, the MB contains neurites of many other neurons. MBONs send dendrites to all MB compartments, are innervated by KCs, and express nicotinic receptors. APL, DPM, octopaminergic neurons all innervate MB lobes. It is true that for all these neurons cell bodies are not located in the vicinity of PAM DANs cell bodies. However, according to the authors themselves they were concerned from “obscure labeled neurites”.

A more selective strategy (e.g., intersectional approaches such as FRT-STOP-FRT) could help ensure PAM DAN-specific labeling.

The conclusion that “These results indicate that memory-inducing PAM cell classes (43, 49, 74) transcriptionally express Dα1” is based only on proximity of the labelled cell bodies to known location of PAM DANs “We detected GAL4-positive somata in the region where PAM DANs are typically located”. I don’t think this is enough.

These concerns are also relevant for the colocalization experiments. The authors use a regular confocal. However, with the dense innervation in the MB and the fact that all neurons in the circuit express nicotinic receptors, this can easily give a false positive result. For this experiment super resolution is required. This is true also for the MiMIC experiments.

The experiments with smOLLAS-tagged Dα1 are not explained anywhere, and it is impossible for the reader to understand that this is cell specific labelling.

The figure quality also makes evaluation challenging:

a. Figure 2A: Where are PAM DANs somata located? PAM DANs should be labeled with anti-TH, or at least their presumed locations should be visually marked.

b. Figures 2B-2C: The picture is not clear - the MB structure, and specifically the gamma lobe, cannot be identified. In addition, the region in Figure 2B, magnified in Figure 2C, should be visually demarcated and explicitly described in the Figure legend.

c. Figure 2D-2F: The picture is not clear. The magnified figures do not seem to be magnification of the low magnification image. In addition, it is unclear how the authors identified axonal terminals/dendrites. Lastly, the detected signals in Figures 2D-2E are not observed in the MB, in contrast to Figures 2A-2C.

2. The manuscript is missing important controls. For example, there is no validation of Dα1 knockdown, not even at the level of the whole brain. There is no attempt to examine acute ACh induced responses. In addition, Dα1 knockdown is performed throughout development. There is no examination that the results do not arise from developmental effects.

3. The authors’ main claim is that Dα1 is required for odors but not sugar induced responses. This is an interesting result. However, the authors do not examine its importance. Are sugar responses mediated by a different neurotransmitter? Are they mediated by the cholinergic muscarinic receptors? Are they mediated by a different nicotinic receptor? Do sugar induced responses have different properties than odor induced responses? Different kinetics? Do they last for longer? Given the relatively limited scope of the results presented, a more thorough examination of these questions would be expected.

The presented data could be improved. The authors claim that Dα1 knockdown reduces odor induced responses but not sugar induced responses. They choose to show the results obtained from alpha1. However, in this brain region only 3-OCT has a significant effect, whereas MCH was not significant, like the sugar.

I guess the authors wanted to show in the same brain region the differential effect. However, this claim is better supported in Figure S1, where across brain regions Sugar response is not affected whereas odor responses are generally affected.

I think it is best to discard current Figure 3 and make Figure S1 new Figure 3. In particular as Figure 3A is identical to the data presented in Figures S1A and S1B. In addition, Figures 3B and 3C are identical to the data presented in Figure S1C.

Scale bar should be located near the traces in Figure S1A and S1B and described in the Figure legend.

4. The authors’ second main claim is that Dα1 is required to selectively gate odor-driven input to support evaluation and selection between competing odor options. However, the authors do not show such gating, nor do they provide evidence on how it is achieved. Is summation of the sugar and odor response required? Is there a change in odor induced signals following conditioning? Is DA release affected?

5. The no-choice result in Figure 4B is supported only by non-significant differences without equivalence testing, effect sizes, confidence intervals, or power analysis to justify an 'indistinguishable' conclusion. Furthermore, this is an easier task than the choice task, especially as high odor concentrations are used (10-fold dilution). Small but meaningful deficits could be missed. It would have been better to examine the no-choice test under more difficult conditions using lower concentrations or odor mixtures.

6. Figure legends lack sufficient detail in several cases, making it difficult to fully interpret the data.

Minor comments:

1. I did not find in Figure 1B the experiments referring to Dα5.

2. There are some errors in the reference list:

a. References 31 and 89 refer to the same manuscript.

b. Reference 98 is wrongly formatted “*Drosophila* ”.

c. Reference 87 was retracted and cannot be cited unless explicitly marking it as retracted and only if not used as evidence as it currently is.

3. I think there is a mistake in Figure 4B protocol bottom. I think the test should be OCT.

4. While I understand the use of log(GCamP/mCherry), this is less intuitive. Perhaps it would be useful to show also DF/F.

5. The behavioral memory index (LI) is not explained in the methods section.

6. The odor generalization experiment is not explained well. The test protocol illustration is unclear. For PI1: the authors presented sugar with the odor, for PI2, they presented the sugar after the odor (and didn’t mention the ISI). It is not clear how this manipulation is related to the question. More importantly, this contrasts with the method explanation: “For the odor generalization protocol (Fig. 4E), flies were trained with an odor for 1 minute in a constant air stream, with or without dried 2M sucrose paper. The conditioned response of the trained flies was subsequently measured.”

7. Line 111: I think it should be “supports” (not “support”).

8. Table 1:

Figure 1A: The Brown-Forsythe test is used to test for the equality of group variances. Was ANOVA conducted? If so, what was the p-value?

The text depicting the statistical test results is not aligned with the table rows. b. ** should be in the row below.

Figures 3B-C: Was a repeated measures 2-way ANOVA conducted?

In general, the main statistical tests used are not explicitly mentioned (e.g., ANOVA, Kruskal-Wallis, t-test, Mann-Whitney), in contrast to the post-hoc tests.

Figure S1 is not listed.

Reviewer #3: This manuscript details the role of nicotinic acetylcholine receptors in dopaminergic PAM neurons in mediating olfactory input during appetitive learning. The story is succinct, the data look good overall, the study includes key controls, and the conclusions/interpretations are supported by the data. The results of the study will add to our understanding of how the circuit functions during reward learning. There are two major aspects of the data presentation that need to be addressed prior to publication.

In figure 2, subcellular colocalization of the Dalpha1 receptor and TH is not clear. There is clear punctate labeling of both. What is the evidence that these are in the same cells? This should be described and pointed out clearly in the figure.

In the physiological/imaging data presented in figures 3B,C and S1, individual responses for both genotypes are intermingled, making it difficult to see the differences. The data should be presented in a more standard format (e.g., box plots for each genotype / time, and/or individual data points + bar graphs [as in the presentation of behavioral data in figure 4]).

**********

**Have all data underlying the figures and results presented in the manuscript been provided?**

Large-scale datasets should be made available via a public repository as described in the *PLOS Genetics*  data availability policy, and numerical data that underlies graphs or summary statistics should be provided in spreadsheet form as supporting information.

Reviewer #1: **No:**  On is missing in Fig.1B

Reviewer #2: None

Reviewer #3: Yes

**********

PLOS authors have the option to publish the peer review history of their article (what does this mean?). If published, this will include your full peer review and any attached files.

If you choose “no”, your identity will remain anonymous but your review may still be made public.

**Do you want your identity to be public for this peer review?**  For information about this choice, including consent withdrawal, please see our Privacy Policy.

Reviewer #1: **Yes:**  Dr. Emmanuel Perisse

Reviewer #2: No

Reviewer #3: No

[NOTE: If reviewer comments were submitted as an attachment file, they will be attached to this email and accessible via the submission site. Please log into your account, locate the manuscript record, and check for the action link "View Attachments". If this link does not appear, there are no attachment files.]

**Figure resubmission:**

**Reproducibility:**

To enhance the reproducibility of your results, we recommend that authors of applicable studies deposit laboratory protocols in protocols.io, where a protocol can be assigned its own identifier (DOI) such that it can be cited independently in the future. Additionally, PLOS ONE offers an option to publish peer-reviewed clinical study protocols. Read more information on sharing protocols at https://plos.org/protocols?utm_medium=editorial-email&utm_source=authorletters&utm_campaign=protocols

# Author comment: Author response to Decision Letter 1

**DOI:** 10.1371/journal.pgen.1012356.r002

5 Sep 2026

Attachment Submitted filename: Yamagata_Rebuttal_Letter_rev.docx

# Peer review: Decision Letter 1

**DOI:** 10.1371/journal.pgen.1012356.r003

**Authors:** Pablo Wappner, Chun Han

15 Oct 2026

Dear Dr Yamagata,

We are pleased to inform you that your manuscript entitled "Nicotinic receptor in dopamine neurons mediates learned odor choice" has been editorially accepted for publication in PLOS Genetics. Congratulations!

Before we can issue the formal final acceptance letter and transfer your files to our production team, there are a few minor remaining items to address. Reviewer 1 has provided a few final comments (appended below) that require textual adjustments. Please address these points in the text and provide a brief point-by-point response letter detailing the changes made. No additional experiments or re-reviews are required; these modifications will be checked directly by the editorial office.

In addition, you will need to complete our formatting changes, which you will receive in a follow up email. Please be aware that it may take several days for you to receive this email; during this time no action is required by you. Please note: the accept date on your published article will reflect the date of this provisional acceptance, but your manuscript will not be scheduled for publication until the required changes have been made.

Once your paper is formally accepted, an uncorrected proof of your manuscript will be published online ahead of the final version, unless you’ve already opted out via the online submission form. If, for any reason, you do not want an earlier version of your manuscript published online or are unsure if you have already indicated as such, please let the journal staff know immediately at plosgenetics@plos.org.

In the meantime, please log into Editorial Manager at https://www.editorialmanager.com/pgenetics/, click the "Update My Information" link at the top of the page, and update your user information to ensure an efficient production and billing process. Note that PLOS requires an ORCID iD for all corresponding authors. Therefore, please ensure that you have an ORCID iD and that it is validated in Editorial Manager. To do this, go to ‘Update my Information’ (in the upper left-hand corner of the main menu), and click on the Fetch/Validate link next to the ORCID field.  This will take you to the ORCID site and allow you to create a new iD or authenticate a pre-existing iD in Editorial Manager.

If you have a press-related query, or would like to know about making your underlying data available (as you will be aware, this is required for publication), please see the end of this email. If your institution or institutions have a press office, please notify them about your upcoming article at this point, to enable them to help maximise its impact. Inform journal staff as soon as possible if you are preparing a press release for your article and need a publication date.

Thank you again for supporting open-access publishing; we are looking forward to publishing your work in PLOS Genetics!

Yours sincerely,

Chun Han, Ph.D.

Academic Editor

PLOS Genetics

Pablo Wappner

Section Editor

PLOS Genetics

Aimée Dudley

Editor-in-Chief

PLOS Genetics

Anne Goriely

Editor-in-Chief

PLOS Genetics

www.plosgenetics.org

BlueSky: @plos.bsky.social

----------------------------------------------------

Comments from the reviewers (if applicable):

Reviewer’s Responses to Questions

**Comments to the Authors:**

**Please note here if the review is uploaded as an attachment.**

Reviewer #1: The authors have address most of my comments, I just have only have minor suggestions to improve the text and the figures:

lines 36, 38, 102 and 250, specify that the knockdown was in PAM DANs

line 114: "In Drosophila, the nAChR repertoire is comparatively small" to what?

line 129: "cholinergic input": in PAM DANs?

Line 161: it will be nice to show the overlap in figure 2G, 2H with little white arrows.

line 173: should the Table S2 be the Table S1 in order of appearance?

line 223: the use of "no choice" is a bit puzzling to me. While I understand the logic compared to the choice between the two odors, but its sounds like there is no choice while they do choose to avoid the tube with odor. Innate preference sounds better.

Line 232: It seems that flies have a strong innate preference towards 4MCH (when compared with 3-OCT). How does that affect learning performances in general?

Line 243: add “learned” in “competing odor alter alternatives.”

Figure S2H and S2G should be inverted to follow the order of the text.

Please consider standardizing the graph Y-axis ranges based on the neighboring subfigures and data measurement type. Specific examples include Figure 1A-1C, where these ranges may be set -0.2 to 0.8, and Figure 3E where -0.4 to -1.2 is most appropriate. Figure S2 would benefit from minor adjustments as well. Editing these graph ranges both allows readers to more quickly compare across conditions and understand the magnitude differences caused by measurement type.

Reviewer #2: The authors have adequately and seriously addressed my comments. I thank them for that. The manuscript is greatly improved and I find the current version of the manuscript suitable for publication.

Reviewer #3: The authors have done a great job in addressing my concerns in the prior round of critiques. I have no further concerns and recommend publication.

**********

**Have all data underlying the figures and results presented in the manuscript been provided?**

Large-scale datasets should be made available via a public repository as described in the *PLOS Genetics*  data availability policy, and numerical data that underlies graphs or summary statistics should be provided in spreadsheet form as supporting information.

Reviewer #1: Yes

Reviewer #2: None

Reviewer #3: Yes

**********

PLOS authors have the option to publish the peer review history of their article (what does this mean?). If published, this will include your full peer review and any attached files.

If you choose “no”, your identity will remain anonymous but your review may still be made public.

**Do you want your identity to be public for this peer review?**  For information about this choice, including consent withdrawal, please see our Privacy Policy.

Reviewer #1: No

Reviewer #2: No

Reviewer #3: No

----------------------------------------------------

**Data Deposition**

If you have submitted a Research Article or Front Matter that has associated data that are not suitable for deposition in a subject-specific public repository (such as GenBank or ArrayExpress), one way to make that data available is to deposit it in the Dryad Digital Repository. As you may recall, we ask all authors to agree to make data available; this is one way to achieve that. A full list of recommended repositories can be found on our website.

The following link will take you to the Dryad record for your article, so you won't have to re‐enter its bibliographic information, and can upload your files directly:

http://datadryad.org/submit?journalID=pgenetics&manu=PGENETICS-D-26-00251R1

More information about depositing data in Dryad is available at http://www.datadryad.org/depositing. If you experience any difficulties in submitting your data, please contact help@datadryad.org for support.

Additionally, please be aware that our data availability policy requires that all numerical data underlying display items are included with the submission, and you will need to provide this before we can formally accept your manuscript, if not already present.

----------------------------------------------------

**Press Queries**

If you or your institution will be preparing press materials for this manuscript, or if you need to know your paper’s publication date for media purposes, please inform the journal staff as soon as possible so that your submission can be scheduled accordingly. Your manuscript will remain under a strict press embargo until the publication date and time. This means an early version of your manuscript will not be published ahead of your final version. PLOS Genetics may also choose to issue a press release for your article. If there’s anything the journal should know or you'd like more information, please get in touch via plosgenetics@plos.org.

# Editor report: Acceptance letter

**DOI:** 10.1371/journal.pgen.1012356.r004

**Authors:** Pablo Wappner, Chun Han

PGENETICS-D-26-00251R1

Nicotinic receptor in dopamine neurons mediates learned odor choice

Dear Dr Yamagata,

We are pleased to inform you that your manuscript entitled "Nicotinic receptor in dopamine neurons mediates learned odor choice" has been formally accepted for publication in PLOS Genetics! Your manuscript is now with our production department and you will be notified of the publication date in due course.

The corresponding author will soon be receiving a typeset proof for review, to ensure errors have not been introduced during production. Please review the PDF proof of your manuscript carefully, as this is the last chance to correct any errors. Please note that major changes, or those which affect the scientific understanding of the work, will likely cause delays to the publication date of your manuscript.

Soon after your final files are uploaded, unless you have opted out or your manuscript is a front-matter piece, the early version of your manuscript will be published online. The date of the early version will be your article’s publication date. The final article will be published to the same URL, and all versions of the paper will be accessible to readers.

For Research Articles, you will receive an invoice from PLOS for your publication fee after your manuscript has reached the completed accept phase. If you receive an email requesting payment before acceptance or for any other service, this may be a phishing scheme. Learn how to identify phishing emails and protect your accounts at https://explore.plos.org/phishing.

Thank you again for supporting PLOS Genetics and open-access publishing. We are looking forward to publishing your work!

With kind regards,

Anitha Samidurai

PLOS Genetics

On behalf of:

The PLOS Genetics Team

Carlyle House, Carlyle Road, Cambridge CB4 3DN | United Kingdom

plosgenetics@plos.org | +44 (0) 1223-442823

plosgenetics.org | Twitter: @PLOSGenetics
